# LncRNA IPW inhibits growth of ductal carcinoma in situ by downregulating ID2 through miR-29c

**DOI:** 10.1186/s13058-022-01504-4

**Published:** 2022-01-25

**Authors:** Ravindra Pramod Deshpande, Sambad Sharma, Yin Liu, Puspa Raj Pandey, Xinhong Pei, Kerui Wu, Shih-Ying Wu, Abhishek Tyagi, Dan Zhao, Yin-Yuan Mo, Kounosuke Watabe

**Affiliations:** 1 Department of Cancer Biology, Wake Forest Baptist Medical Center, Winston-Salem, NC 27157 USA; 2 Nirogy Therapeutics, Framingham, MA 01702 USA; 3 Frederick National Laboratory for Cancer Research, Frederick, MD 21701 USA; 4 Department of Pharmacology and Toxicology, Cancer Institute, University of Mississippi Medical Center, Jackson, MS 39216 USA

**Keywords:** LncRNA IPW, DCIS, ID2, miR-29c, Toyocamycin

## Abstract

**Background:**

Ductal carcinoma in situ (DCIS) of breast is the noninvasive lesion that has propensity to progress to the malignant form. At present, it is still unknown which lesions can potentially progress to invasive forms. In this study, we aimed to identify key lncRNAs involved in DCIS growth.

**Methods:**

We employ disease-related lncProfiler array to identify IPW in specimens of DCIS and matching control samples and validate the observations in three DCIS-non-tumorigenic cell lines. Further, we examine the mechanism of IPW action and the downstream signaling in in vitro and in vivo assays. Importantly, we screened a library containing 390 natural compounds to identify candidate compound selectively inhibiting IPW low DCIS cells.

**Results:**

We identified lncRNA IPW as a novel tumor suppressor critical for inhibiting DCIS growth. Ectopic expression of IPW in DCIS cells strongly inhibited cell proliferation, colony formation and cell cycle progression while silencing IPW in primary breast cells promoted their growth. Additionally, orthotropic implantation of cells with ectopic expression of IPW exhibited decreased tumor growth in vivo. Mechanistically, IPW epigenetically enhanced miR-29c expression by promoting H3K4me3 enrichment in its promoter region. Furthermore, we identified that miR-29c negatively regulated a stemness promoting gene, ID2, and diminished self-renewal ability of DCIS cells. Importantly, we screened a library containing 390 natural compounds and identified toyocamycin as a compound that selectively inhibited the growth of DCIS with low expression of IPW, while it did not affect DCIS with high IPW expression. Toyocamycin also suppressed genes associated with self-renewal ability and inhibited DCIS growth in vivo.

**Conclusion:**

Our findings revealed a critical role of the IPW-miR-29c-ID2 axis in DCIS formation and suggested potential clinical use of toyocamycin for the treatment of DCIS.

**Supplementary Information:**

The online version contains supplementary material available at 10.1186/s13058-022-01504-4.

## Background

Ductal carcinoma in situ (DCIS) refers to the cancerous state in which breast epithelial cells undergo abnormal proliferation but are still restricted within the ducts and lobules [1]. DCIS represents up to 25% of newly diagnosed cases of breast cancer, and about 40% of DCIS considered to progress to invasive carcinoma if untreated [2]. DCIS is commonly treated by surgical resection (lumpectomy and mastectomy) followed by adjuvant radiotherapy [3], which has raised the scientific debate on potential overtreatment of DCIS. Because more than half of DCIS patients are not likely to have progressive invasive cancer [4], it is important to differentiate patients who are over treated and would have benefited from minimal therapeutic intervention [5]. Even with the advent of molecular profiling, it is still largely unknown why certain DCIS lesions transition to invasive phenotype, while other remains dormant [6]. This is mainly due to the unpredictable biology, functional heterogeneity and dearth of competent preclinical model system to study DCIS. Therefore, it is critical to decipher fundamental biological pathways and regulatory processes associated with DCIS and its progression to invasive phenotype.

Long-noncoding RNAs (lncRNAs) belong to the class of noncoding RNAs greater than 200 nucleotides in length. Initially considered as the transcriptional noises, lncRNAs have been increasingly appreciated for their involvement in a range of cellular processes [7]. To date, lncRNAs are overwhelmingly catalogued in different databases for their roles in various physiological as well as pathological processes [8]. LncRNAs are predominantly found to be localized in the nucleus and impacts gene expression in multiple ways including epigenetic modification of transcriptionally regulatory regions and activating or blocking access of transcriptional factors to these regulatory site of the genes [9]. LncRNAs also regulate gene expression by sponging miRNAs and thereby scavenging its inhibitory function [10]. However, the functional role and underlying mechanism of action of lncRNAs in DCIS are yet to be determined. Therefore, identification and functional annotation of lncRNA(s) that drives the pathological growth of DCIS are urgently needed.

In this study, we performed screening of lncRNAs in DCIS and matching normal breast tissues to identify lncRNAs involved in DCIS. We found IPW as a novel tumor-suppressive lncRNA in DCIS. We then elucidated the functional role of IPW in suppressing DCIS stem cell population via regulation of ID2 expression. Mechanistically, IPW epigenetically promoted the expression of miR-29c, which further targeted ID2 to suppress self-renewal ability of DCIS. Overall, our results suggest that the decrease in IPW expression serves as a critical driver of DCIS growth and orchestrates miR-29c-dependent suppression of cancer stem cell. Importantly, we identified a natural compound, toyocamycin, that selectively targets IPW-low DCIS cells and inhibits DCIS growth in vivo.

## Results

### LncRNA IPW is downregulated in DCIS

**Fig. 1:**
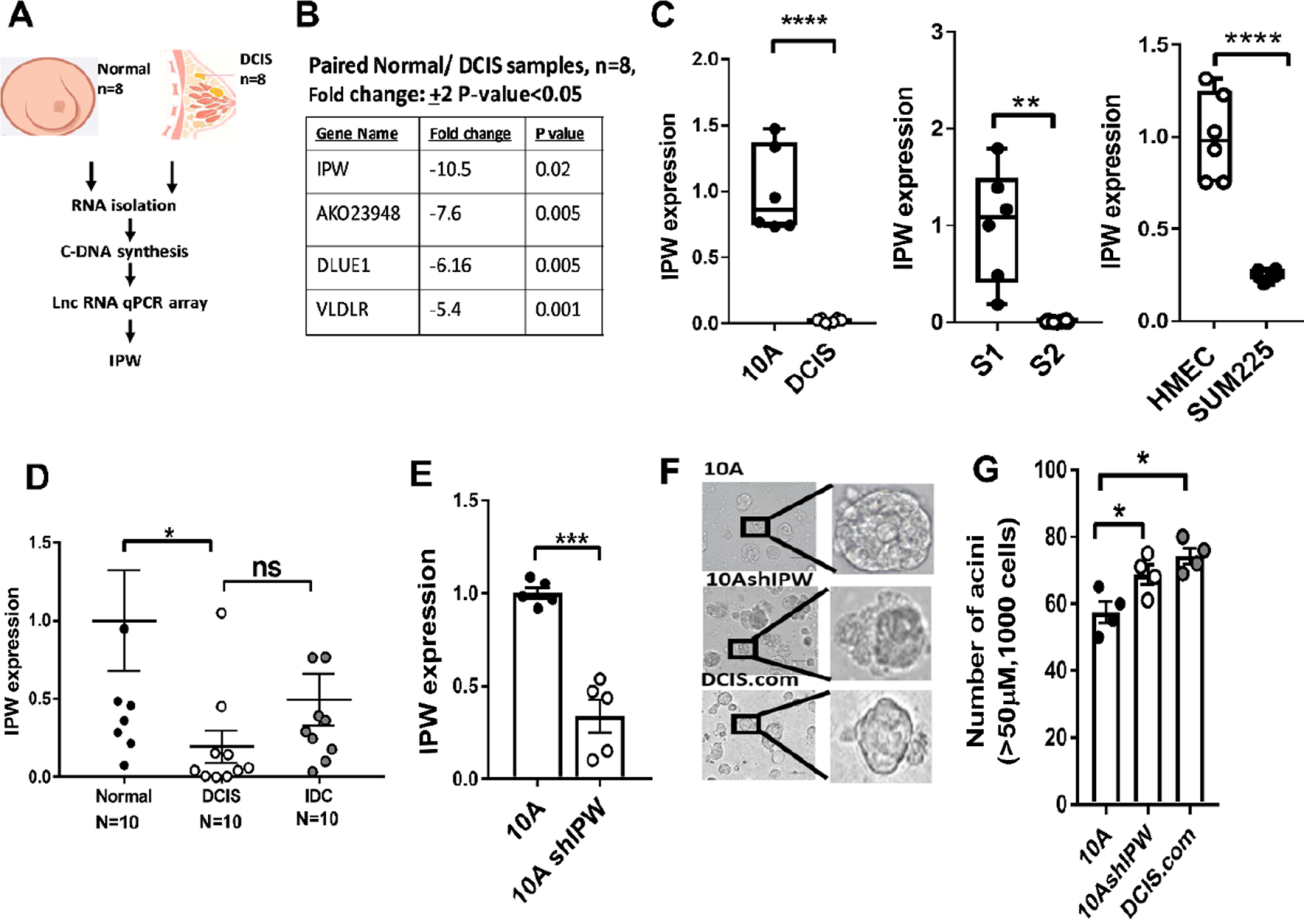
LncRNA IPW is downregulated in DCIS. **A** Schematic diagram of lncRNA profiling from DCIS and adjacent normal tissues. DCIS and adjacent normal tissues (*n* = 8 each) were homogenized and total RNAs were extracted from these samples. Expression of LncRNAs in SBI lncRNA profiler array was examined by qRT-PCR. **B** LncRNAs that were differentially expressed (fold change + 2) with statistical significance (*p*-value < 0.05) are listed in the table. **C** IPW expression was examined in 10A and DCIS (left panel), S1 and S2 (middle panel) and HMEC and SUM225 (right panel) by qRT-PCR (*n* = 5/group). Unpaired two-tailed Student’s *t* test was performed for statistical comparison. **D** IPW expression was examined by qRT-PCR in RNA extracted from normal, DCIS and IDC formalin-fixed paraffin-embedded slides by microdissection (*n* = 10/group). One-way ANOVA with Tukey multiple comparison post hoc test was performed. **E** IPW expression was examined in MCF10A (control) and MCF10A-shIPW cells (*n* = 5/group) by qRT PCR. Unpaired two-tailed Student’s t test was performed. **F** 3D culture was performed in Matrigel-coated 96 well plate. MCF10A, MCF10A-shIPW and DCIS.com cells were reconstituted in 10% Matrigel and seeded into Matrigel-coated plate. Representative bright-field images were acquired by microscopy at days 10–13 (*n* = 4/group). Scale bar 50 µm. **G** Acini formed in Figure F that were > 50uM in diameter were counted at days 10–13. Data are represented as mean + S.E.M (*n* = 4/group). (*p < 0.05, **p < 0.01, ****p < 0.0001). ns: not significant

To identify differentially expressed lncRNAs in DCIS, we isolated total RNA from eight paired normal and DCIS tissues and performed qRT-PCR for disease-related lncRNAs (Fig. 1A). Four lncRNAs were found to be downregulated with significant *p* value (< 0.05) and fold change higher than 5 (Fig. 1B). LncRNA IPW (Imprinted in Prader–Willi syndrome) was most significantly downregulated in DCIS as compared to normal tissues (Additional file 1: Fig. S1A). We further validated the expression of top four lncRNAs in three pairs of non-tumorigenic breast epithelial cells and DCIS cell lines: MCF10A and DCIS.com, S1 and S2, HMEC and SUM225. IPW was observed to be most significantly downregulated in DCIS cell lines when compared to its non-tumorigenic controls (Fig. 1C, Additional file 1: Fig. S1B–D). Therefore, IPW was selected for subsequent analysis. To further substantiate the clinical relevance of IPW in DCIS, we isolated RNAs from normal ducts, DCIS lesions and invasive ductal carcinoma (IDC) from patient specimens in formalin-fixed paraffin-embedded (FFPE) sections by microdissection (*n* = 10/group). We observed that IPW was significantly downregulated in DCIS and IDC when compared to the normal samples (Fig. 1D). IPW expression was then verified in cell lines representing non-tumorigenic (MCF10A), DCIS (DCIS.com) and IDC (MCF10CA). Similar to the clinical samples, IPW expression was significantly decreased in DCIS.com (Additional file 1: Fig. S1E), suggesting that IPW plays a role in DCIS formation. As changes in morphology of ducts are early events of carcinoma initiation [6, 11, 12], we examined whether IPW was involved in regulating duct size and morphology by performing acini formation assay in 3D culture. We silenced IPW expression in MCF10A cells using short hairpin RNAs (shRNAs) (Fig. 1E), and cultured cells in Matrigel-embedded culture plates (Fig. 1F). We observed that the average size of the ducts formed by cells silenced for IPW was significantly larger with irregular morphology and in higher number when compared to the parental MCF10A (Fig. 1G). This result suggests that IPW plays a functional role in the maintenance of duct integrity, and loss of IPW expression triggers DCIS transition. Collectively, our results strongly indicate that IPW is a clinically relevant lncRNA downregulated in DCIS and plays a critical role in its initiation.

### *IPW suppresses DCIS growth *in vitro* and *in vivo*:*

**Fig. 2:**
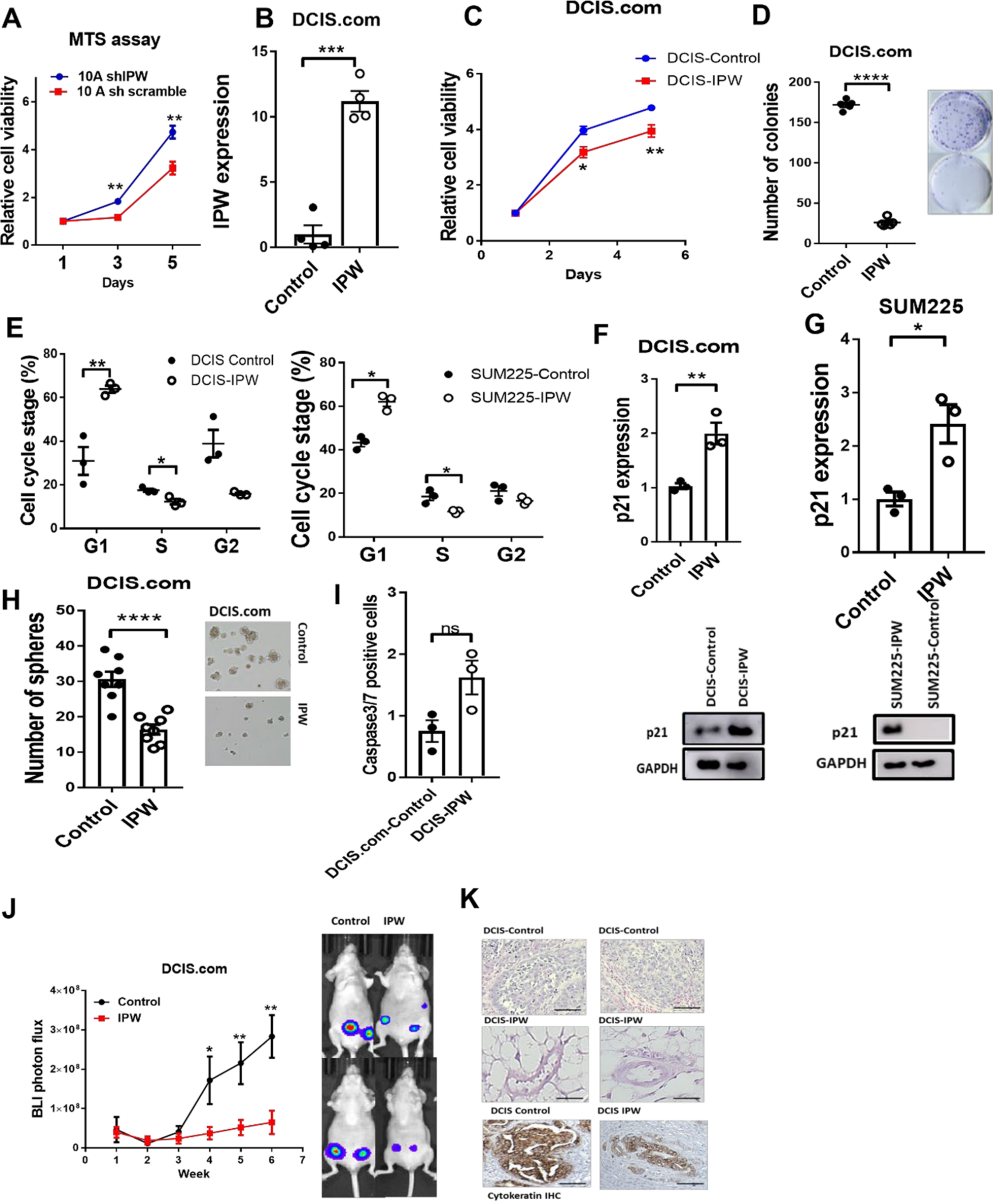
IPW inhibits DCIS growth *in vitro* and *in vivo*. **A** MCF10A and 10AshIPW cells were seeded in a 96-well plate (5000 cells/well, *n* = 6/group), and cell viability was evaluated at days 1, 3 and 5 by MTS assay. **B** LncRNA IPW or empty vector was ectopically expressed in DCIS.com cell line using lentivirus, and IPW expression was examined in DCIS (control) and IPW-expressed DCIS.com cells (*n* = 4/group) by qRT-PCR. **C** DCIS.com cells were transduced with either empty vector or IPW. 5000 cells were seeded in 96-well plate (*n* = 6/group) and relative cell viability was examined by MTS assay at days 1, 3 and 5. **D** DCIS.com and DCIS-IPW cells were seeded in six-well plates (*n* = 500 cells/well, *n* = 5/group), and number of colonies were counted at day 10. All data were analyzed by unpaired two-tailed Student’s *t* test. **E** DCIS.com (left panel) and SUM225 (right panel) cells transduced with empty vector or IPW were subjected to cell cycle analysis using flow cytometry. % of cells at G1, S and G2 phase are shown (*n* = 3/group). Cells were synchronized and stained with PI followed by visualization by FACS. The data were analyzed using ANOVA and Tukey’s multiple comparison test. **F**, **G** DCIS.com and SUM225 wells were transduced with either empty vector or IPW were examined for p21 mRNA and protein expression. p21 mRNA expression was evaluated qRT-PCR (*n* = 3/group; upper panels) and protein expression by western blot (bottom panels). The statistical inference for qRT-PCR was calculated by unpaired two-tailed Student’s *t* test. **H** DCIS.com cells were ectopically expressed with either empty vector or IPW by lentivirus transduction and evaluated by mammosphere assay. 1000 cells were seeded/well in mammosphere media (*n* = 8/group). Number of spheres were calculated at D5 under microscope. Scale bar 50 µm **I** 20,000 cells were seeded in 24-well plate for 3 days. At the end of incubation, cells were incubated with and 4 µM of Caspase3/7 green detection reagent for 1 h at 37^0^C. Then, cells were rinsed with PBS to wash off the unbound reagent followed by trypsinization and analysis by flow cytometry. **J** DCIS.com (control) and DCIS-IPW cells were implanted in the mammary fat pad of nude mice (*n* = 5, 1 × 106 cells/mouse), and tumor growth was monitored by bioluminescence (BLI). Representative IVIS images are shown in right panel. Animals were killed when the tumor volume is 1000mm3 or when the animals became morbid. **K** H&E staining was performed for tumors isolated at the endpoint in Study (upper and middle panels). Tumor sections were also stained for human cytokeratin by immunohistochemistry (lower panel). Representative images are shown. Scale bar 100 µm. All statistical inference between the two points was evaluated by unpaired two-tailed Student’s t test. Data are represented as mean + S.E.M. (*p < 0.05, **p < 0.01, ***p < 0.001, ****p < 0.0001)

To gain further insight into the role of IPW in DCIS growth, we examined the growth of MCF10A and MCF10A-shIPW cell lines in vitro. As shown in Fig. 2A, we found that the growth rate of IPW-silenced MCF10A cells was significantly increased when compared to the parental MCF10A. We then overexpressed IPW in DCIS.com (Fig. 2B), SUM225 (Additional file 1: Fig. S2A) and S2 (Additional file 1: Fig. S2F) cells by lentivirus-mediated transduction and studied the effect of IPW in cell growth and colony formation ability of these cells. We found that IPW expression significantly suppressed cell proliferation and colony-forming ability of these cells, demonstrating its strong tumor suppression function in vitro (Fig. 2C, D). Similar results were observed in SUM225 (Additional file 1: Fig. S2B, C) and S2 (Additional file 1: Fig. S2G, H) cell lines. Additionally, ectopic expression of IPW stalled cells in the G1 phase of cell cycle, thereby inhibiting cell cycle progression in DCIS.com, SUM225 (Fig. 2E) and S2 (Additional file 1: Fig. S2I) cell lines. IPW overexpression also upregulated p21 mRNA and protein in DCIS.com and SUM225 cell line (Fig. 2F, G). As our group and others have previously demonstrated the role of cancer stem-like cells (CSC) in DCIS growth [13–15], we examined the effect of IPW in DCIS stemness. We found that IPW expression significantly decreased sphere formation ability in DCIS.com (Fig. 2H), SUM225 (Additional file 1: Fig. S2D, E) and S2 (Additional file 1: Fig. S2J, K) cells. However, we observed that ectopic IPW expression (Additional file 1: Fig. S2L) did not alter wound healing (Additional file 1: Fig. S2M) and migration ability of DCIS.com, MCF-7 and 10CA (Additional file 1: Fig. S2N) cells. Similarly, IPW expression did not affect caspase 3/7 expression indicating no marked change in cellular death (Fig. 2I). Furthermore, to address the effect of IPW expression in DCIS growth, we implanted luciferase-labeled DCIS.com cells transduced with either empty vector or IPW in the mammary fat pad of nude mice and examined tumor growth every week by bioluminescence (BLI). The size of lesions of the mice implanted with DCIS-IPW cells was significantly smaller as measured by BLI photon flux index (Fig. 2J). Furthermore, hematoxylin and eosin (H&E) staining of the tumor section revealed that ducts formed by DCIS-IPW tumors had lower cellularity and normal-like structures when compared to control tumors (Fig. 2K upper and middle panels). We also confirmed that these ducts were derived from DCIS.com-implanted cells, and not from the mouse mammary tissue, by positive staining of normal-like ducts using human cytokeratin antibody (Fig. 2K lower panel). Together, these results suggest that IPW expression inhibits DCIS growth by inducing cell cycle arrest and reducing stemness property. We found that IPW expression did not decrease in IDC compared to DCIS, indicating that IPW downregulation is an early event in tumorigenesis. However, when we segregated breast cancer patients based on IPW expression through KM plotter (*n* = 2032), (Additional file 1: Fig. S2O) dataset, we found that high IPW expression is correlated with significantly longer overall survival of patients suggesting that IPW is a clinically relevant lncRNA in cancer. In connection with these observations, we examined the p21, ID2 and miR-29c RNA expression in IDC lines 10CA and MCF7 ectopically expressed with either empty vector (control) or IPW. We observe that p21 and miR-29c were upregulated while ID2 was suppressed in 10CA (Additional file 1: Fig. S2P) and MCF7 (Additional file 1: Fig. S2Q) upon IPW overexpression. These results consolidate the tumor-suppressive role of IPW in DCIS and IDC. However, as the molecular drivers and signaling in IDC are very complex and subtype-dependent, further experiments are warranted to consolidate the mechanism of IPW in IDC.

### miR-29c is downregulated in DCIS and is controlled by IPW

**Fig. 3:**
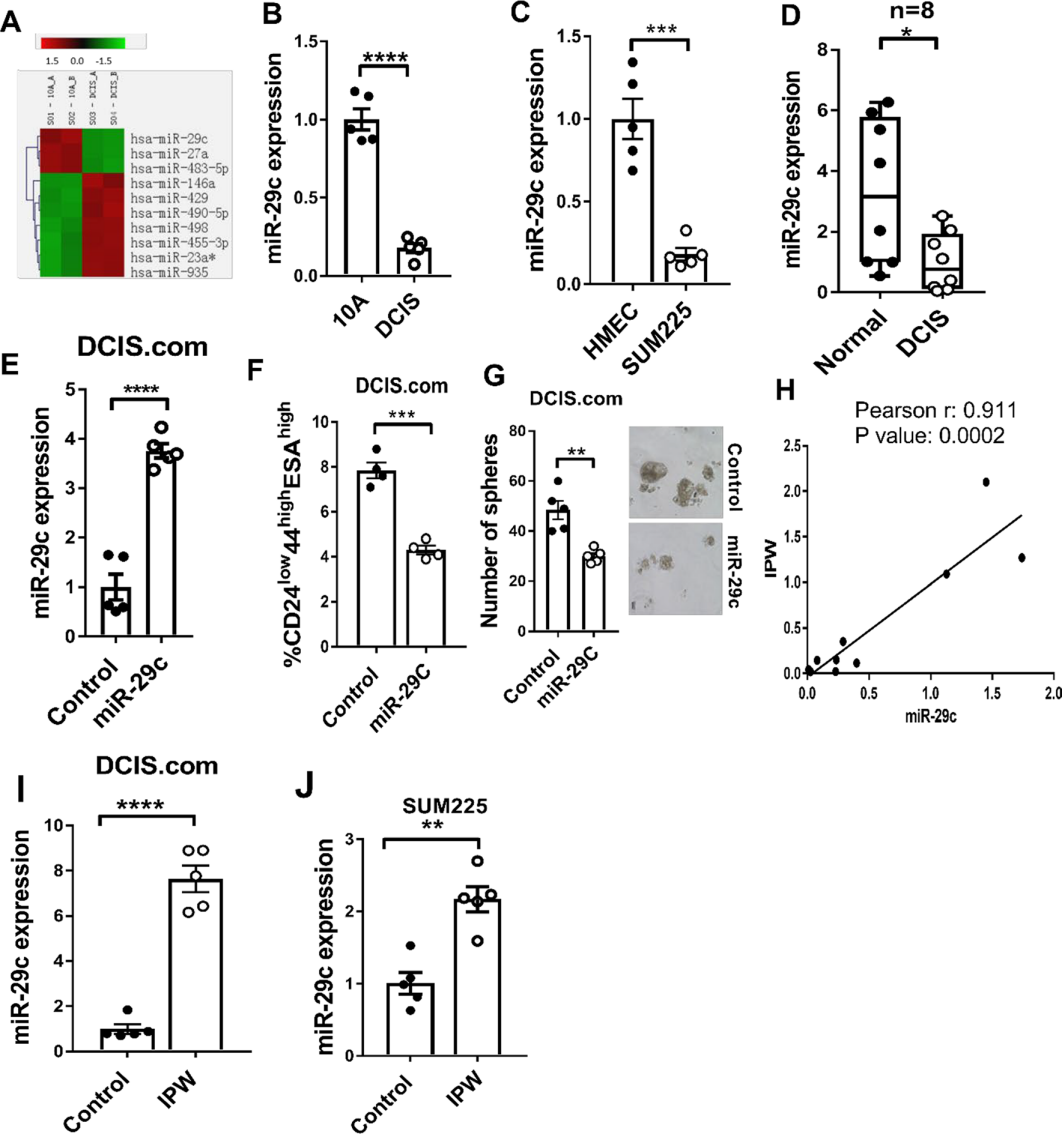
miR-29c is downregulated in DCIS and is controlled by IPW. **A** RNAs were isolated from MCF10A and DCIS.com cells and subjected to Affymetrix microRNA array. Differentially expressed miRNAs based on *p*-value (< 0.05) were plotted as a heat map. **B** miR-29c expression in MCF10A and DCIS.com was quantified by qRT-PCR (TaqMan probes, *n* = 5/group). **C** miR-29c expression was examined in HMEC and SUM225 cells by TaqMan qRTPCR (*n* = 5/group). **D** miR-29c expression was examined in DCIS and matching normal tissues used for Fig. 1A by TaqMan qPCR (*n* = 8/group). **E** DCIS.com cells were transduced with either empty vector or miR-29c-expressing vector and miR-29c expression was examined by TaqMan qRT-PCR (*n* = 5/group). **F** DCIS.com cells were transduced with either empty vector or miR-29c-expressing vector. Tumor initiating stem cell (CD24^low^44^high^ESA^high^) population in was quantified by flow cytometry (*n* = 4/group). **G** DCIS.com (control) and DCIS.com-miR-29c cells were seeded in ultra-low attachment plate supplemented with mammosphere media (1000 cells/well, *n* = 5/group). Number of spheres were calculated at day 5 and visualized under the microscope. **H** IPW and miR-29c expression were examined in RNA samples isolated from DCIS tumor sections by qRT-PCR (*n* = 10/group). Correlation between IPW and miR-29c is shown. The degree of correlation was inferred from Pearson *r* and *p* value. **I**, **J** miR-29c expression was examined by TaqMan-based qPCR in empty vector or IPW expressed DCIS.com (**I**) and SUM225 (**J**) cells (*n* = 5/group). miR-361-5p was used as internal control. Statistical inference unless otherwise specified was determined by unpaired two tailed Students t test. Data is represented as mean + S.E.M. (*p < 0.05, **p < 0.01, ***p < 0.001, ****p < 0.0001)

Micro RNAs (miRNAs) are known to be involved in regulating multiple cellular processes in breast cancer [16, 17] including regulation of the downstream signaling to lncRNA, often through direct transcriptional control or sponge effect [18, 19]. In an attempt to uncover differentially expressed miRNAs in DCIS, we performed miRNA microarray in MCF10A and DCIS.com cell line. In this screening, we found that miR-29c was the most downregulated miRNA in DCIS.com as compared with MCF10A (Fig. 3A). The decrease in miR-29c expression was further verified by TaqMan qPCR in DCIS.com and SUM225 cells compared to MCF10A and HMEC, respectively (Fig. 3B, C). We then examined miR-29c expression in the same set of matching DCIS and adjacent normal tissue samples used for initial screening (Fig. 1A). miR-29c was significantly downregulated in DCIS samples compared to its the paired normal tissues (Fig. 3D). Furthermore, ectopic miR-29c expression significantly decreased CSC marker-positive population and sphere formation ability of DCIS.com cells (Fig. 3E-G). Importantly, we found a strong positive correlation between IPW and miR-29c expression in DCIS samples (Fig. 3H), indicating a potential regulatory interconnection between IPW and miR-29c. Therefore, to study whether IPW regulated miR-29c expression, we examined miR-29c expression in DCIS.com and SUM225 cells with ectopic expression of IPW. As shown in Fig. 3I, J, IPW significantly upregulated miR-29c expression in DCIS.com and SUM225 cells. On the other hand, ectopic expression of miR-29c did not affect IPW expression, suggesting that IPW is the upstream regulator of miR-29c (Additional file 1: Fig. S3). These results indicate that miR-29c plays a tumor-suppressive role in DCIS and its expression is controlled by IPW.

### IPW upregulates miR-29c expression by enhancing H3K4 trimethylation

**Fig. 4:**
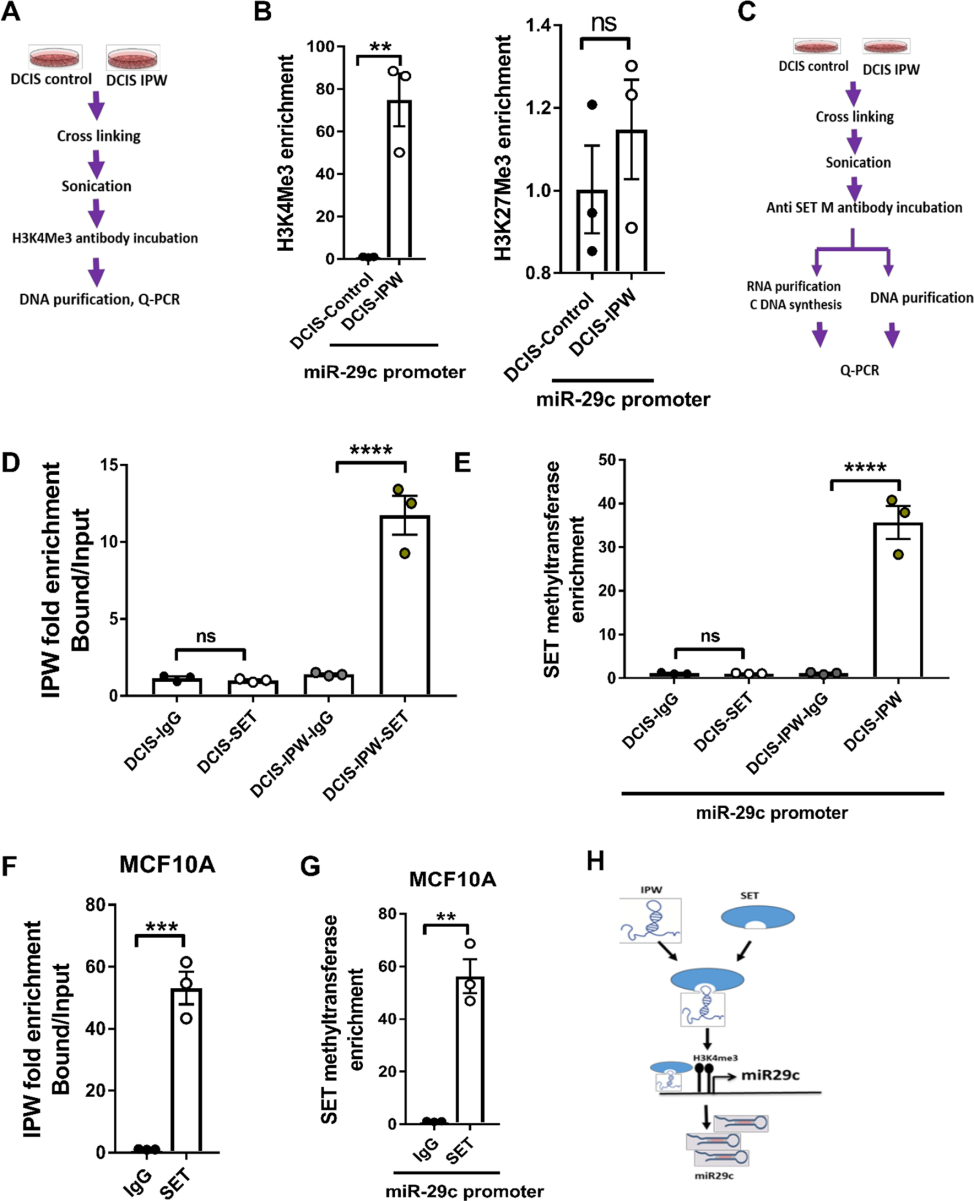
IPW upregulates miR-29c expression by enhancing H3K4 trimethylation. **A** Experimental scheme of ChIP assay. **B** ChIP assay was performed in DCIS-control and DCIS-IPW cells using specific antibodies to H3K4me3- (left panel) and H3K27me3- (right panel). The enrichment of miR-29c promoter region was examined by qRTPCR (*n* = 3/group). **C** Experimental scheme of SET methyl transferase immunoprecipitation assay. **D** DCIS (control) and DCIS-IPW cells were immunoprecipitated by IgG or SET-methyltransferase antibody followed by isolation of RNA and cDNA synthesis. Enrichment of IPW was examined by qRT-PCR (*n* = 3/group). **E** DNA was isolated from DCIS-control and DCIS-IPW cells immunoprecipitated with IgG or SET methyltransferase (as shown in C) followed by quantitation of miR-29c promoter enrichment by qRT-PCR (*n* = 3/group) **F** MCF10A cells were immunoprecipitated by IgG or SET methyltransferase antibody followed by isolation of bound RNAs. Enrichment of IPW in the IgG- or SET methyltransferase-bound RNAs was examined by qRT-PCR. **G** MCF10A cells were immunoprecipitated by IgG or SET methyltransferase antibody followed by isolation of bound DNAs. Enrichment of miR-29c promoter region in the IgG- or SET methyltransferase-bound DNAs was examined by qRT-PCR. Statistical inference between two groups was determined by unpaired two-tailed Student’s t test and data are represented as mean + S.E.M. **H** Hypothesis figure showing IPW interaction with SET methyltransferase and locating the later to miR29c promoter region. This results in enhanced H3K4me3 leading to increased miR-29c expression. (*p < 0.05, **p < 0.01, ***p < 0.001, ****p < 0.0001). ns: not significant

We then sought to decipher how IPW controlled miR-29c expression. LncRNAs are known to epigenetically regulate histones via posttranslational modifications, thereby affecting gene expression [20, 21]. Therefore, we utilized the UCSC genome browser to examine regulatory histone modifications marks in the promoter region (140 bp upstream of TSS) of miR-29c. We found that histone 3 lysine 4 trimethylation (H3K4me3), known for activating gene expression [22–24], was highly enriched near the transcription start site (TSS) of miR-29c (Additional file 1: Fig. S4). To verify the presence of H3K4me3 in miR-29c TSS, we performed chromatin immunoprecipitation (ChIP) in DCIS and DCIS-IPW cells using H3K4me3 antibody (Fig. 4A). A significant enrichment of H3K4me3 was observed in DCIS-IPW cells (Fig. 4B left panel) in miR-29c promoter region. However, the enrichment of histone 3 lysine 27 trimethylation (H3K27Me3) was not evident in the same region (Fig. 4B right panel), suggesting that H3K4me3 enrichment in the miR-29c promoter region is specifically enhanced by IPW. As H3K4Me3 is known to be mediated by SET methyltransferase [25, 26], we immunoprecipitated SET methyltransferase in DCIS.com and DCIS-IPW cells (Fig. 4C) followed by isolating RNA and DNA bound to the protein. In the RNA fraction immunoprecipitated by SET methyltransferase, we found IPW to be significantly enriched in DCIS-IPW compared to parental DCIS.com cells, suggesting that SET methyltransferase interacts directly to IPW and this interaction is dependent on the level of IPW expressed by cells (Fig. 4D). On the other hand, we examined the miR-29c promoter region in the DNA fraction immunoprecipitated by SET methyltransferase. As shown in Fig. 4E, strong enrichment of SET methyltransferase was evident in the miR-29c promoter region of IPW-expressing cells. Similarly, the enrichment in IPW RNA and miR-29c promoter bound to SET methyltransferase was evident in MCF10A cells (Fig. 4F,G). Together, these results indicate that IPW positively regulates miR-29c expression through SET methyltransferase-mediated H3K4me3 enrichment in miR-29c promoter region (Fig. 4H).

### miR-29c suppresses self-renewal ability of DCIS by targeting ID2

**Fig. 5:**
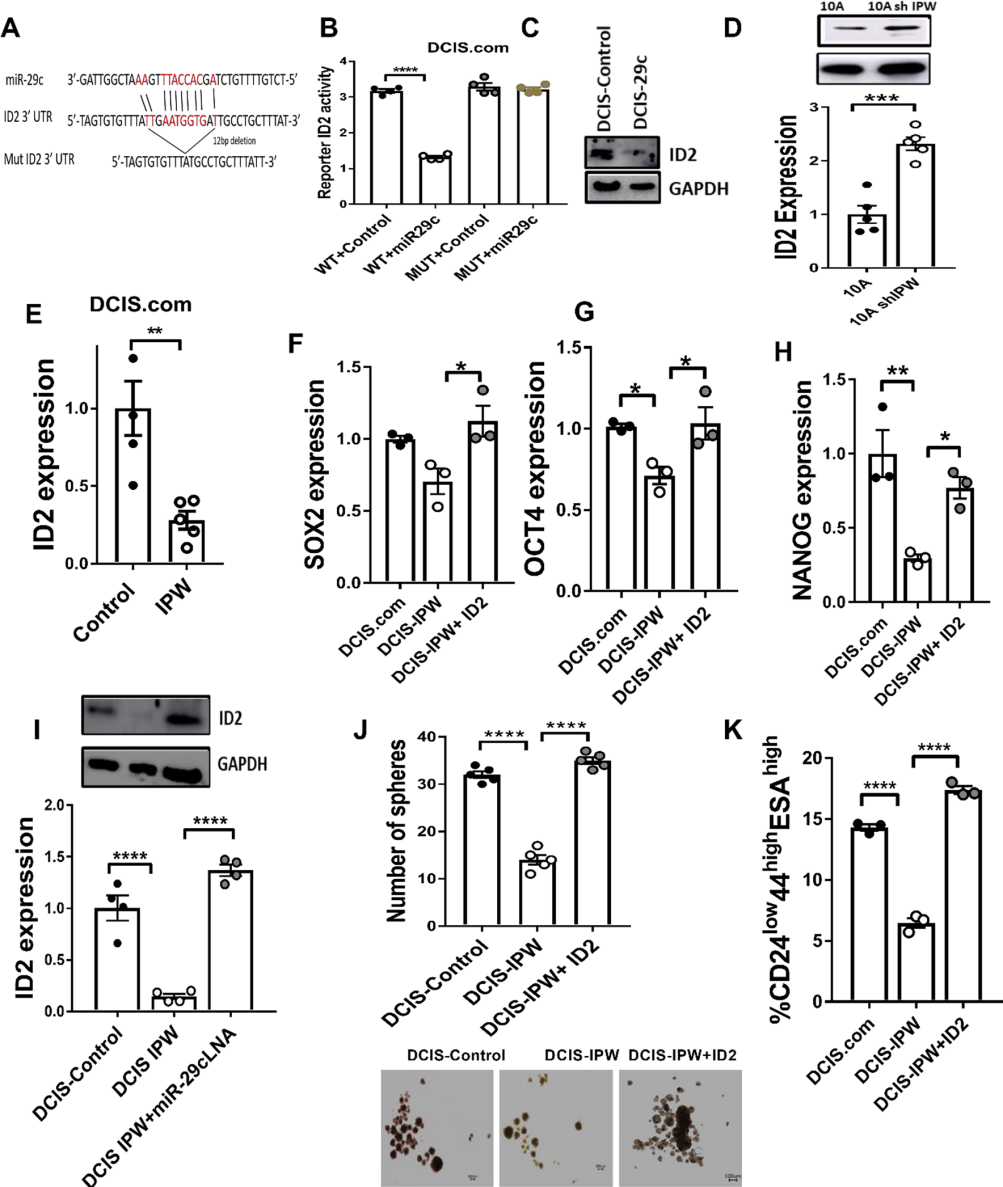
miR-29c suppresses self-renewal ability of DCIS by targeting ID2. **A** Figure showing the potential binding site of miR-29c on ID2 mRNA 3’UTR. The 12 base miR-29c binding region (colored in red) was deleted from the ID2 3’UTR. **B** DCIS.com cells were co-transfected with wild-type ID2 3’UTR luciferase reporter and miR-29c-expressing vector or empty vector, and luciferase activity was measured using luminometer. Similarly, DCIS.com cells were co-transfected with mutated ID2 3’UTR luciferase reporter (region of mutation are red colored in Figure A) and miR-29c-expressing vector or empty vector, and luciferase activity was quantified using luminometer (*n* = 4/group). Statistical inference between the groups was determined by ANOVA with Tukey multiple comparison post hoc test. **C** DCIS.com cells transduced with either empty or miR-29c-expressing vector and cell lysates were prepared. ID2 protein expression was examined in the lysates by western blot and imaged by Amersham imager. **D** ID2 expression was evaluated in 10A(control) and 10A shIPW cells by qRT-PCR (lower panel) and western blot (upper panel). For qRT-PCR (*n* = 5/group), statistical inference was calculated using unpaired two-tailed Student’s t test and represented as mean + SEM. **E** Effect of IPW on ID2 mRNA expression was examined by qRT-PCR in DCIS.com cells transduced with pSIN-IPW or empty vector followed by RNA isolation. Actin was used as internal control (*n* = 5/group, statistical inference was made using unpaired two-tailed Student’s t-test and represented as mean + SEM). **F**–**H** SOX2 (F), OCT4 (G) and NANOG (H) mRNA expression was examined in DCIS.com (control), DCIS-IPW and DCIS-IPW cells ectopically expressed with ID2 (*n* = 3/group) by qRT-PCR actin used as internal control. One-way ANOVA with Tukey multiple comparison post hoc test was performed for statistical comparison. **I** DCIS (control), DCIS-IPW and DCIS-IPW were transfected with miR-29c-locked nucleic acid (LNA). ID2 expression was evaluated by qRT-PCR (*n* = 4/group; lower panel) and western blot (upper panel). One-way ANOVA with Tukey multiple comparison post hoc test was performed for statistical comparison of qRT-PCR results. **J** DCIS.com (control), DCIS-IPW and DCIS-IPW cells ectopically expressed with ID2 were seeded on ultra-low attachment 96-well plate (1000 cells/well, *n* = 5/group), and number of spheres formed by these cells were counted at day 5. **K** Tumor-initiating stem cell population (CD24lowCD44highESAhigh) was examined in DCIS.com(control), DCIS-IPW and DCIS-IPW cells transduced with ID2-expressing vector by flow cytometry (*n* = 3/group). One-way ANOVA with Tukey multiple comparison post hoc test was performed for statistical comparison and data are represented as mean + S.E.M. (*p < 0.05, **p < 0.01, ***p < 0.001)

Because IPW epigenetically regulated the expression of miR-29c, we sought to determine the downstream target of miR-29c. Serendipitously, we found a 12-bp miR-29c binding region in the 3’ UTR of ID2, a gene that we previously reported to strongly promote self-renewal of DCIS (Fig. 5A) [13]. We then mutated the 12-bp miR-29c binding site in ID2 3’UTR as shown in Fig. 5A and performed the promoter reporter assay for ID2 expression. We found that while miR-29c expression significantly decreased reporter activity, the mutation of the binding site in the 3’ ID2 UTR completely rescued the reporter activity in DCIS.com cells (Fig. 5B). In addition, ectopic expression of miR-29c strongly suppressed ID2 protein expression in DCIS.com cells (Fig. 5C). We then examined the effect of IPW on ID2 expression. Silencing of IPW in MCF10A cells significantly augmented ID2 mRNA and protein levels (Fig. 5D). Conversely, IPW expression in DCIS.com and SUM225 cells significantly reduced ID2 expression (Fig. 5E and Additional file 1: Fig. S5A). Similarly, ID2 expression was found to be decreased in tumors formed by DCIS.com cells with IPW expression (from animals in Fig. 2I) when examined by immunohistochemistry (Additional file 1: Fig. S5B, C). Additionally, the expression of transcription factors known to promote tumor stemness (SOX2, OCT4 and NANOG) [27, 28] were significantly downregulated in DCIS cells expressing IPW and rescued when ID2 was expressed (Additional file 1: Fig. S5D, Fig. 5F–H). Next, we examined whether miR-29c is crucial to mediate the downstream effect of IPW on ID2 expression and stem cell population. The miR-29c-mediated inhibition of ID2 expression was rescued when we transfected miR-29c locked nucleic acid (LNA) in DCIS.com cells expressing IPW (Fig. 5I). Similarly, ID2 expression in DCIS-IPW cells rescued the number and size of spheres (Fig. 5J) and tumor initiating stem cell population (Fig. 5K). We then examined the impact of IPW expression on self-renewal in DCIS.com cells. It was previously demonstrated by Al-Hajj et al. that CD44 + cells have the potential to self-renew and form mammary tumors in mice [29]. In corroboration with this observation, we isolated CD24^low^CD44^high^ESA^high^—DCIS.com and DCIS-IPW cells by magnetic-assisted cell sorting (MACS) and performed mammosphere formation assay. We observed that the number of spheres formed by DCIS-IPW cells was significantly decreased in first- and second-generation mammosphere culture (Additional file 1: Fig. S5E).Together, these results indicate that IPW controls the DCIS self-renewal by targeting ID2 expression through miR-29c.

### Toyocamycin selectively inhibits low IPW-expressing DCIS

**Fig. 6:**
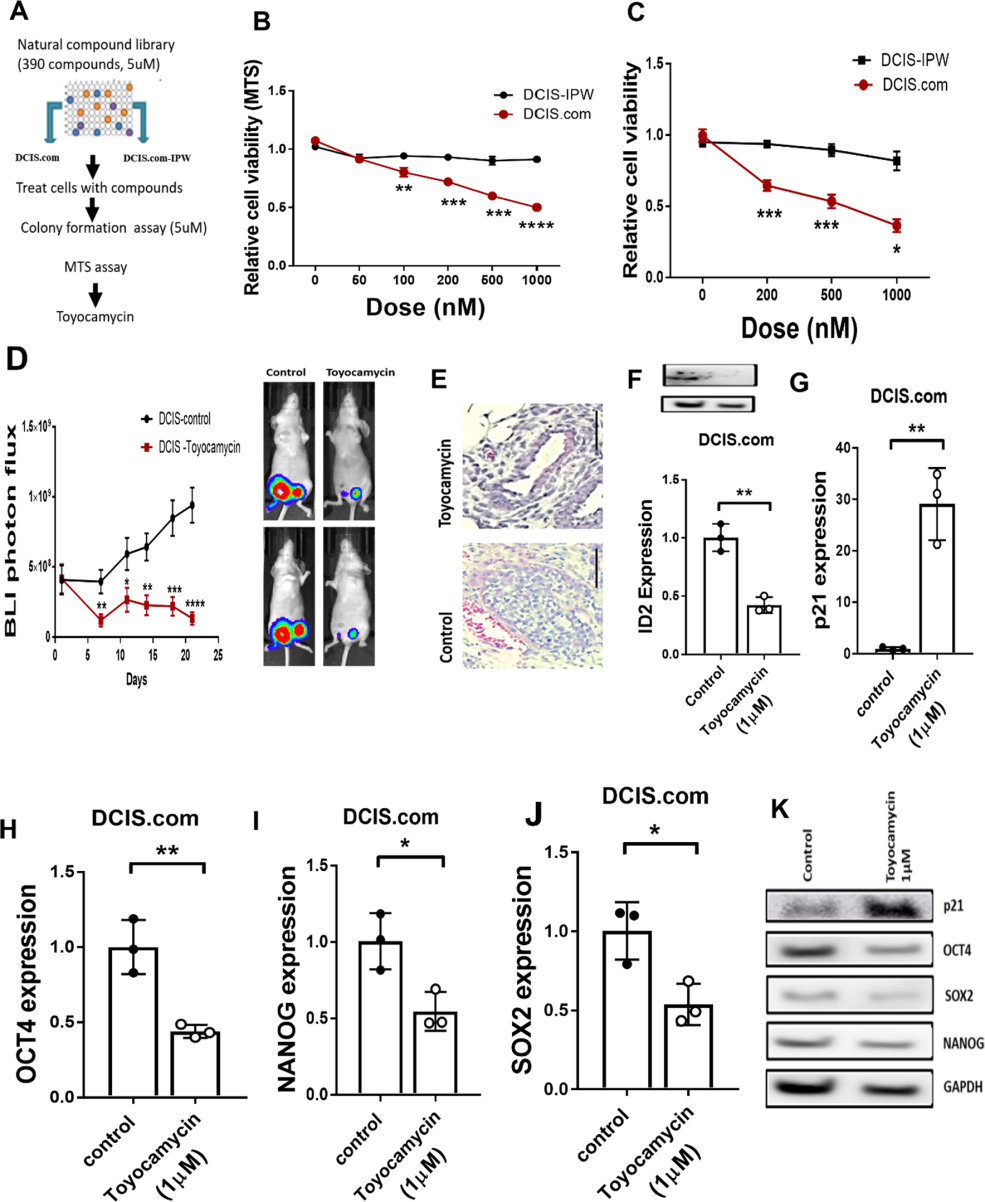
Toyocamycin selectively inhibits low IPW-expressing DCIS. **A** Flowchart of the natural compound library screening. DCIS.com and DCIS-IPW cells were seeded in 96-well plates (5000 cells/well) and treated individually with 390 natural compounds at 5uM final concentration. Cells were treated for 48 h, and colonies were stained with crystal violet. Then, the stain was dissolved in 10% acetic acid, and absorbance was obtained at 595 nm absorbance in spectrophotometer. **B** DCIS.com and DCIS-IPW cells were treated with toyocamycin at indicated concentrations for 48 h, and relative cell viability was examined by MTS assay (5000 cells/well, *n* = 3/group). Statistical inference was made by unpaired two-tailed Student’s t test. **C** DCIS.com and DCIS-IPW cells (500 cells/well, *n* = 3/group) were treated with toyocamycin at indicated concentrations for 10 days in 6-well dish. Colonies formed at day 10 were stained with crystal violet, and the dye was dissolved in 10% acetic acid followed by measuring absorbance at 595 nm. Statistical inference was calculated by unpaired two-tailed Student’s t test. **D** Luciferase-labelled DCIS.com cells (1 × 10^6^ cells/mice, *n* = 6/group) were implanted into nude mice through m.f.p injection, and they were treated with either toyocamycin at 2 mg/kg body weight or control (DMSO) once a week. Animals were randomized before starting drug treatment. Tumor growth was monitored by IVIS bioluminescence. Representative animal pictures from control and toyocamycin treated group are shown on right side. **E** H&E staining was performed in tumors from DCIS implanted mice in Figure D. Representative image from each group is shown. Scale bar 100 µm. **F:** DCIS.com cells were treated with control or toyocamycin (1uM) for 24 h, and ID2 mRNA and protein expression was examined by qRT-PCR (*n* = 3/group) and western blot, respectively. Actin was used as internal control. Statistical inference was made by using Student’s t test. **G**–**J** DCIS.com cells were treated with control (DMSO) or toyocamycin (1uM) for 24 h and p21 (**G**), OCT4 (**H**), SOX2 (**I**) and NANOG (**J**) mRNA expression was examined by qRT-PCR (*n* = 3/group). Actin used as internal control and statistical inference was made by using two-tailed Student’s t test. **K** DCIS.com cells were treated with either control (DMSO) or toyocamycin (1uM) for 24 h and lysates were prepared. p21, OCT4, SOX2 and NANOG protein expressions were determined by western blot. Actin expression was used as a loading control. Statistical inference between two groups was determined by unpaired two-tailed Student’s *t* test, and data are represented as mean + S.E.M. (*p < 0.05, **p < 0.01, ***p < 0.001, ****p < 0.0001)

As cancer stem cells are known to drive transition of DCIS to invasive carcinoma, a potential strategy for treating DCIS and decreasing its probability of transitioning to aggressive carcinoma is to selectively target DCIS stem-like tumor-initiating cells. Our results strongly indicated that downregulated IPW in DCIS promoted self-renewal ability of DCIS stem cells. Therefore, we aimed to identify compounds that can selectively inhibit DCIS cells with a low IPW expression. We screened a natural compound library containing 390 drugs in DCIS cells and IPW-expressing DCIS cells by colony formation and MTS assay (Fig. 6A, Additional file 1: Fig. S6A). We found that toyocamycin significantly inhibited the relative cell viability and colony-forming ability of IPW-low DCIS cells at low concentration of 100 nM (Fig. 6B, C). Next, we examined the in vivo efficacy of toyocamycin on DCIS. We found that toyocamycin significantly suppressed the tumor growth in DCIS.com in animals with dosed at 2 mg/kg (Fig. 6D, E). Furthermore, no significant weight loss or change in alanine aminotransferase (AST) activity was observed in the blood of toyocamycin-treated mice (Additional file 1: Fig. S6B, C). Next, we sought to determine the mechanism of action of toyocamycin. We found that toyocamycin significantly downregulated the expression of ID2 and stem cell-associated transcription factors OCT4, SOX2 and NANOG (Fig. 6F–K) but did not affect IPW and miR-29c levels **(**Additional file 1: Fig. S6D, E). The effect of toyocamycin on p21, ID2, OCT4, SOX2 and NANOG expression was also verified in the tumor sections by immunohistochemistry (Additional file 1: Fig. S6F). In addition, we confirmed that the stained tumor tissues were derived from the implanted cells by staining tumor sections with human cytokeratin antibody (Additional file 1: Fig. S6F). Toyocamycin has been shown previously shown to regress the Ewing sarcoma [30] and multiple myeloma [31] by targeting the ER stress response genes. However, we observed no significant difference in expression of XBP, JNK, CHOP and ATF4 in DCIS.com cells treated with toyocamycin (Additional file 1: Fig. S6G–J). We also verified that toyocamycin does not affect the proliferation ability of non-tumorigenic cell lines MCF10A and HMEC (Additional file 1: Fig. S6K). These results suggest that toyocamycin specifically suppresses IPW-low DCIS cells by targeting ID2 and can potentially be used to treat patients with DCIS to avoid the current overtreatment practice.

## Discussion

IPW is a paternally encoded lncRNA, and a lack of its expression plays a significant role in the pathology of neurodevelopmental disorder known as Prader–Willi syndrome (PWS) [32]. However, there has been no report till date on the role of IPW in tumor biology. One previous study examined the expression of IPW in germ-, bladder- and embryonal cancer cell line and showed that IPW is expressed by both alleles suggesting the loss of maternal imprinting in these lines. The authors also found that IPW expression remains unchanged in differentiated Tera-2 cells when compared to non-differentiated cell line and concluded that IPW has a “housekeeping” role during the cell growth [33]. In contrast, our study strongly indicates the tumor-suppressive role of IPW in DCIS. To our knowledge, this is the first study reporting tumor-suppressive function of IPW and mechanism of action of IPW in tumor pathology. Furthermore, we have demonstrated the therapeutic effect of targeting DCIS cells with low IPW expression, which strongly indicates that the role of IPW in DCIS is beyond housekeeping capacity. Interestingly, previous reports studying a cohort of 1160 patients reported a higher incidence of leukemia in PWS patients (eight PWS patients had leukemia compared to expected number of 4.8). Specifically, the incidence of myeloid leukemia cases in PWS patients was significantly higher [34]. In addition, another group studying 56 PWS patients estimated increased risks for testicular cancer, lymphatic leukemia and breast cancer [35]. Of note, our results also indicate that the knockdown of IPW induces morphological changes to the acini formed by breast epithelial cells, suggesting its potential role in early carcinoma initiation. Although larger cohort study is required to validate the effect of IPW in cancer initiation, our finding does indicate its tumor-suppressive effect in early carcinoma of the breast. It is also plausible that the inhibitory function of IPW is tumor type- and context-specific.

LncRNAs orchestrate endogenous signaling in pathological conditions in myriad ways including sponging of miRNAs and regulation of gene expression through histone modifications [36, 37]. One predominant mechanism of lncRNAs includes epigenetic alterations of histones by adding or removing posttranslational modifications such as methylation and acetylation at specific histone residues [38]. IPW was previously reported to epigenetically regulate expression of genes encoded by DLK1-DIO3 locus in PWS. IPW interacted with G9 methyltransferase to impart repressive H3K9me3 modification on the chromatin that led to the suppression of DLK1-DIO3 locus genes [32]. On the contrary, we provide mechanistic evidence that IPW is recruited to the regulatory region of miR-29c and enrich activating-type histone methylation (H3K4Me3) [39, 40] to promote miR-29c expression in DCIS. Our results also indicate that IPW physically binds and recruits SET methyltransferase, an enzyme known to catalyze H3K4me3 [25], to the upstream region of miR-29c. Consistent to our findings, previous reports have also shown that lncRNAs augment H3K4me3 and activate protein coding genes proximal to H3K4me3-enriched chromatin [41, 42]. While there is adequate mechanistic evidence showcasing the miRNA competing or "sponge” effect of lncRNAs [43], there is a scarcity of reports on miRNA expression promoting the effect of lncRNAs. Therefore, our findings have discovered a novel epigenetic mechanistic axis of lncRNA IPW in promoting miR-29c expression in DCIS.

There are raising numbers of evidence indicating the prominence of cancer stem cells (CSC) in perpetuating growth of solid tumors, and the property of progressive growth and therapeutic refractiveness in DCIS is often attributed to cancer stem cell component [14, 44]. Our group and others have previously demonstrated the role of cancer stem-like cells in DCIS growth and progression [13, 45–47]. In the present study, we provide evidence on the CSC inhibitory effect of IPW through ID2 downregulation. ID2 is a transcription factor with helix–loop–helix DNA binding domain [48]. In our previous study, we found that ID2 promotes CSC self-renewal and its expression was critical for the transition of DCIS to invasive ductal carcinoma (IDC). In this study, we found that IPW significantly decreased ID2 expression in DCIS through miR-29c. Our results indicate that abrogation of miR-29c expression by locked nucleic acid targeting miR-29c in IPW-expressed DCIS cells strongly rescued the inhibitory effect of IPW on self-renewal ability. miR-29c was previously reported to play a tumor-suppressive role by targeting key pathways in cancer and CSC population [49, 50]. Furthermore, ectopic miR-29c expression sensitizes cancer cells to paclitaxel chemotherapy [51]. Similarly, ID2 expression in cancer is also correlated with self-renewal ability and resistance to chemotherapy [52, 53], suggesting the significance of miR-29c and ID2 axis in cancer. Our findings suggest that downregulation of IPW-miR-29c axis is critical for the maintenance of CSC in DCIS, which emphasizes the importance of IPW-miR-29c in early carcinogenesis and its progression.

We have shown that toyocamycin is a compound that suppresses the growth of DCIS cells with low IPW expression *in vitro* and *in vivo.* In particular, toyocamycin was found to exert a significant effect in preventing tumor growth in animals. Previous studies have shown that toyocamycin inhibits the growth of Ewing sarcoma [30] and multiple myeloma [31] without any obvious side effects, thus consolidating our evidence. To extrapolate the translational significance of pharmacological approach, we propose that DCIS patients should be screened for IPW expression, and fivefold lower expression cutoff could be set to expose patients to competent treatments aimed to prevent progression to IDC. As toyocamycin was shown to inhibit the ID2-dependent cancer stem cell enrichment, we speculate that this compound could be of immense value for the clinical use where DCIS patients are generally over treated to prevent its progression to the invasive forms.

## Conclusion

**Fig. 7:**
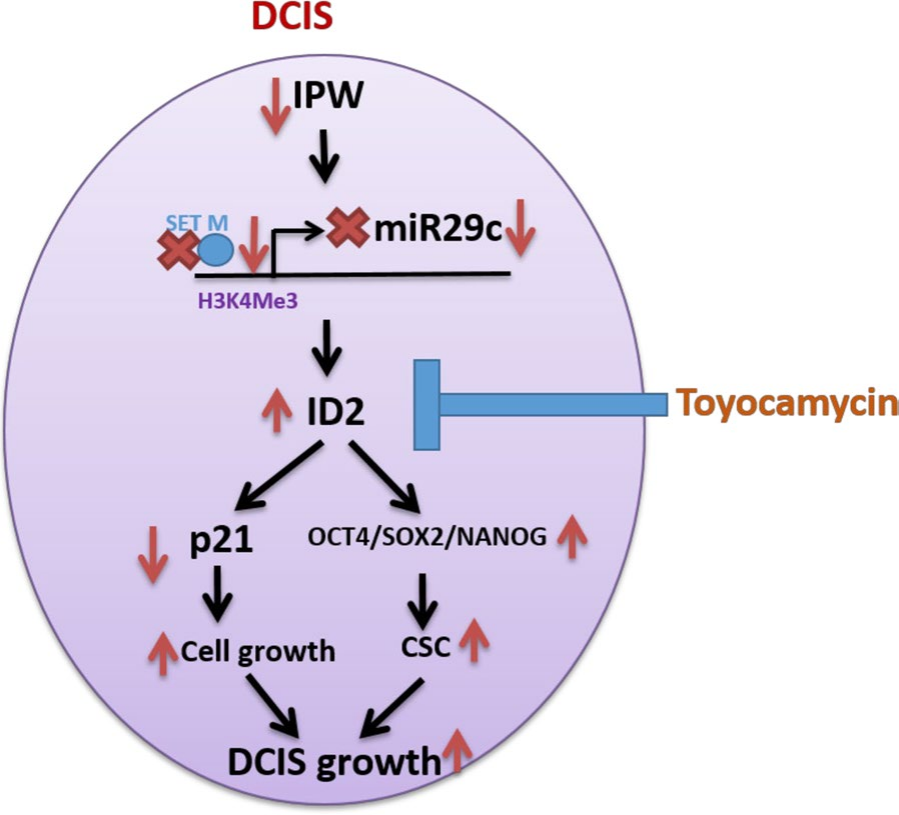
The proposed model of the downstream components of IPW in regulating DCIS growth and mechanism of toyocamycin action

In conclusion, we identified IPW as a novel lncRNA possessing tumor-suppressive and carcinoma initiation inhibitory functions. On a mechanistic scale, IPW promoted miR-29c expression by inducing H3K4 trimethylation in the regulatory chromatin region of miR-29c. Furthermore, IPW-mediated miR-29c upregulation suppressed the ID2 expression and reduced the tumor initiating stem cell population. The inhibitory effect of IPW in DCIS growth was also verified in vivo using an immunocompromised mice model. Finally, by performing natural compound library screening, we identified that toyocamycin selectively kills IPW-low-expressing DCIS cells and could be a candidate compound to treat DCIS patients to prevent overtreatment (Fig. 7).

## Materials and methods

### Cell lines and reagents

DCIS.com was purchased from Asterand, Inc. S1 and S2 were purchased from Millipore Sigma, USA. Human mammary epithelial cell line (HMEC) was purchased from Lonza. SUM225 was a kind gift from Dr. Fariba Behbod, University of Kansas Medical Centre. MCF10A and HEK293T were purchased from ATCC. MCF 10A was cultured in DMEMF-12 (Gibco) supplemented with 5% horse serum (Thermo, cat no.: 16050130), 100 ng/ml cholera toxin (Sigma, cat no.: C8052), 20 ng/ml EGF (Peprotech, cat no.: AF-100-15), 100ug/ml insulin (Sigma, cat no.: 10516-5ML) and 0.5ug/ml hydrocortisone (Sigma, cat no.: H0888). DCIS.com, and MCF7 were cultured in RPMI medium, while S2 was cultured in DMEM medium supplemented with 10% FBS (Sigma), Penicillin (100 Units/ml) and Streptomycin (100ug/ml). HMEC and MCF10A were cultured in human mammary epithelial growth medium (Lonza). SUM225 was cultured in DMEM-F12 medium supplemented with 5%FBS, Penicillin (Gibco)- 100 Units/ml, Streptomycin (Gibco)- 100ug/ml, Hydrocortisone-1ug/ml, insulin-5ug/ml and HEPES-1 mM. SYBR green was purchased from Bio-Rad (Cat no:1725124). Sphere media were prepared by mixing 100 ml of DMEM/ F12 media with 2 ml of B27 supplement (Invitrogen, cat no.:17504), 100ul of 20ug/ml EGF (Peprotech, cat no. AF-100-15), 40ul of 10 mg/ml Insulin (Sigma, cat no.: 10516-5ML). For bioluminescence imaging, DCIS.com cells were transduced with lentivirus containing firefly luciferase. IPW expression was silenced by using shRNAs purchased from Abmgood (Cat no.: 2504209).

### Three-dimensional cell culture

MCF10A, MCF10AshIPW and DCIS.com cells were cultured on regular 96-well plastic dishes (Fisher) in 10% Matrigel with phenol red as described before [54] In brief, the culture plates were coated with Matrigel and allowed to solidify for 20 min at 37 °C. Afterward, the cells were trypsinized and counted by hemocytometer. 1000 cells were suspended in 60 ul of medium with Matrigel (10%) and added on Matrigel-precoated wells drop wise. Cells were allowed to attach for 30 min at 37 °C. Afterward, 60ul of medium was added on the cells making final concentration of Matrigel to 5%. The medium was changed every 48 h, and cells were observed for acini between 10 and 12th day.

### Wound healing and cellular migration assay

For wound-healing assay, cells were seeded and grown till confluence. Then, a wound is made with 200 µl tip and cells were washed with PBS. Afterward, complete media were added and cellular migration was monitored at 0 h, 12 h and 24 h. The diameter of wound was calculated by measuring the distance covered by migrated cells at 6 points around a scratch and average was derived. Each experiment was performed in triplicate, and results were represented as mean + SEM. For transwell migration assay, 5000 cells were seeded on the upper part of cell culture inserts coated with growth factor-reduced Matrigel (8.0 µM pore size, BD) and number of migrated cells were calculated after 24 h by fixing in 4% formalin followed by crystal violet staining. Cells were grown in medium without serum in the chamber, while media with 5% serum were added in the bottom as chemoattractant.

### Disease-related lncRNA profile array

Disease-related lncRNA plate was procured from SBI (SBI, cat no. RA900A-1). The PCR array plate contained 83 lncRNAs and 11 housekeeping genes. The manufacturer’s instructions were followed. Briefly, total RNA was isolated from the DCIS and matching normal tissue with the use of Direct-zol RNA miniprep kit (Zymo, cat no: R2061) as per the manufacturer’s instructions. The tissue samples were procured from the tissue core at Wake Forest School of Medicine. All samples were pathologically confirmed and were completely anonymized. RNA purity was verified with Nano Drop spectrophotometer (Fisher Scientific). 1ug of RNA was used for cDNA synthesis with the use of Script CDNA synthesis kit (Bio-Rad, cat no 1708890) and diluted to 100 µl as per the instructions. This cDNA was further used for SYBR green-based qPCR for 40 cycles. The reaction conditions were: 50 °C-2 min, 95 °C-10 min, 95 °C-15 s, 60 °C-1 min. The results were analyzed by relative quantification. The fold change in gene expression was calculated as per the instructions relative to control samples (https://systembio.com/wp-content/uploads/Disease_related_Profiler_WEB-1.pdf).

### Cloning

IPW construct was provided by Dr. Benvenisty [32]. IPW from pEGF N1 IPW was then sub-cloned in pSIN lentiviral plasmid vector. 4.5 kb of IPW was amplified with the help of primers: F: GC ACTAGT ccaaatcata gatcaagata tt, R: CTA GGATCC ttacacaggtttaagaat, Tm: 60^0^ C, 35 cycles. Amplified PCR product was purified from the gel using gel extraction kit (Qiagen, Cat no: 28506). The extracted IPW was cloned in pSIN-Pur plasmid (Addgene, cat no: 16579). Restriction enzymes were purchased from Thermo Fischer Scientific (SpeI-FD1254, BamH1- FD0054). Plasmid-expressing miR-29c was purchased from Origene. Five shRNAs spanning IPW were procured from Abmgood. A mixture of five individual shRNAs were used in to generate lentivirus in HEK293T cells and used for infecting cell lines (DCIS.com, SUM225, S2, MCF10A). For infection, cells with 60% confluence were seeded and incubated with virus particles for 8 h. Afterward, the virus solution was replaced with complete media and cells were grown for 48 h. Puromycin selection (10ug/ml) was employed to positively select the shRNAs expressing cells.

### Cell proliferation, colony and sphere formation assay

To assay cellular proliferation, 1000 cells were seeded in each well of 96-well plate and incubated for 1,3,5 and 7 days. 20ul of CellTiter 96 aqueous one solution cell proliferation assay (MTS) solution (Promega G3582) was added to cells and incubated for 90 min. The 490-nm absorbance was then quantified in the plate reader. For colony formation assay, 500 cells were seeded in each well of six-well plate. Media were replaced after every 3 days. Cells were fixed with cold methanol for 20 min at room temperature and stained with crystal violet (0.5% w/v). Colonies were counted under stereomicroscope. Alternatively, colonies were dissolved in 10% acetic acid and readouts were obtained at 595 nm. For sphere assay, 1000 cells were seeded in ultra-low attachment plates (Corning, cat no.: 3473) in sphere media. The spheres > 40 µm were quantified and imaged at day 5 by Olympus microscope.

### Isolation of tumor initiating stem-like cells and mammosphere assay

DCIS initiating stem like cells were isolated by using magnetic-assisted cell sorting (MACS) column as described previously [55]. Briefly, DCIS.com and DCIS-IPW cells were trypsinized and stained with biotin-conjugated anti-CD24 (Miltenyi, cat no.: 130-095-951) and APC-conjugated anti-CD44 microbeads and incubated on ice for 15 min. After washing the samples with PBS, CD24-negative cells sorted by incubating with anti-biotin microbeads (Miltenyi Biotec). The flushed fraction of cells was then mixed with anti APC microbeads (Miltenyi Biotec) and biotin-conjugated anti-ESA antibody (StemCell, Cat. no.:17653) for 15 min on ice. CD44 and ESA positive fractions were collected and cultured in mammosphere media in ultra-low attachment plates. For serial passaging, mammospheres were first centrifuged and washed with PBS. Cells were then dissociated with trypsin and neutralized with FBS. The disaggregated spheres were then counted and seeded in ultra-low attachment plate with mammosphere media and number of spheres was calculated on day 5.

### Caspase 3/7 assay

CellEvent Caspase3/7 green detection reagent was used to examine the cell death induced upon ectopic IPW expression in DCIS.com cells. 20,000 cells were seeded in 24-well plate and grown for 72 h. Afterwards, media was removed, cells were washed with PBS and incubated with 4 µM of Caspase3/7 green detection reagent for 1 h at 37^0^C. At the end of incubation, cells were washed twice with PBS to wash off the reagent, trypsinized and Caspase3/7 positive cells were quantified by flow cytometry.

### Cell cycle analysis

For cell cycle analysis, IPW or empty vector-infected cells were seeded in 6-well plates. Cells were synchronized by growing in FBS-deprived media overnight. Cells were then cultured for 48 h in FBS-supplemented culture media. Cells were trypsinized, resuspended in 300ul of PBS and fixed in 700ul of cold EtOH (100%) for 2 h at -20 °C, followed by 2X PBS washing, propidium iodide (PI) staining (1 mg/ml) and RNase A treatment (1ug/ml) for 30 min at room temperature. Stained cells were analyzed by flow cytometry (Accuri-BD).

### Real-time PCR

Total RNA was extracted from the cells with the use of Direct-zol RNA miniprep kit (Zymo, cat no.: R2061) as per the manufacturer’s instructions. Purity of RNA was examined by Nano Drop spectrophotometer, and 200 ng of RNA was used for cDNA synthesis by iScript CDNA synthesis kit (Bio-Rad, cat no 1708890). cDNA was amplified with the selected pair of forward and reverse primers using Bio-Rad CFX-Connect device by SYBR green-based amplification. IPW F: 5’-AGGGCAGTGCGTATTTGAAG-3’, IPW R: 5’-GGACAGTTTTGCTTCCCTAG-3’, ID2 F: 5′ -TCAGCACTTAAAAGATTCCGTG-3′, ID2 R: 5′-GACAGCAAAGCACTGTGTGG-3’, β-Actin F:5’-TGAGACCTTCAACACCCCAGCCATG-3’, β-Actin R: 5’-CGTAGATGGGCACAGTGTGGGTG-3’ XBP F: 5’- GGCCGACGGGACCCCTAAAG-3’, R: 5’- CCTCAGCGCCTTCTCCTCGG-3’, JNK F: 5’- CGGTCTGGCCAGGACTGCAG-3’, R: 5’- GCCCATGCCAAGGATGACCTCG-3’ ATF4 F: 5’- GACCAGTCGGGTTTGGGGGC-3’, R: 5’- GACTGACCAACCCATCCACAGCC-3’, CHOP F:5’- GCTGGGAGCTGGAAGCCTGG-3’, R: 5’- GCTGGTTCTGGCTCCTCCTCAG-3’. miR-29c amplification was carried out using TaqMann probes (Applied bio system-479229), and miR-361 (Applied biosystem-478056) was used as internal control. Briefly, RNA was isolated from the cells as mentioned and purity was confirmed using Nano Drop spectrophotometer (Fisher Scientific). Ten nanograms of RNA was used for cDNA synthesis. miRNA cDNA synthesis was carried out using TaqMan advanced miRNA cDNA synthesis kit (Applied Biosystems, cat no.: A28007) as per manufacturer’s instructions. Quantitative PCR was performed with TaqMan Fast Advanced Master Mix (2X) with total reaction volume of 15 µl. Reaction conditions were followed as instructed for 7500 and 7500 Fast systems for 40 cycles. The results were analyzed by relative quantification method. All qRT PCR results were normalized with respective non-tumorigenic/control experimental set. miR-29c LNA probes (miRCURY-YI04105460) were used to silence the endogenous miR-29c expression. For LNA transfection, 200,000 cells per well were seeded in 12-well plate. LNA probe (2uM) was mixed with lipofectamine RNAiMax for 5 min in opti-MEM medium and added to the cells.

### Chromatin immunoprecipitation and SET methyltransferase pulldown

Cells were grown at 80% confluence in 10-cm dish. EpiTect ChIP One Day kit (Qiagen) was used for immunoprecipitation experiment, and the manufacturer’s instructions were followed. Briefly, cells were cross-linked with 1% formaldehyde and resuspended in cell harvesting buffer containing protease inhibitor cocktail by scraping. Then, the suspension was subjected to sonication (Covaris) to generate average size of 750 bp. The lysate was centrifuged to 14,000 *g* × 10 min at 4 °C to pellet debris. Afterward, the chromatin was precleared with protein A beads for 50 min at 4 °C with rotation. The chromatin was incubated with 10ug of anti-H3K4me3 and -H3K27Me3 antibodies (Millipore) at 4 °C with rotation. Before this, 10ul of solution was removed to use as IP fraction. After incubation, the cross-links were reversed in combination with ChIP grade proteinase K in high salt condition as given in the manual. DNA was purified and used for qRT PCR with given pairs of primers: F: 5’-CAGGGGGGAAATCAGAATATC-3’, R: 5’-ATTTGACCATGGGCTTGCGG-3’. The data were represented as enrichment relative to input. Magna Nuclear RIP kit (Millipore, cat no: 17-10521) was used for SET methyltransferase pulldown. The original protocol was slightly modified to isolate both DNA and RNA. In brief, the cells were cross-linked, and chromatin was sheared by homogenization. The DNA digestion step was avoided. SET antibody (5ug, CST, cat no 61702) and IgG (5ug, provided with kit) were loaded on magnetic beads and immunoprecipitated overnight at 4 °C. Cross-links were then reversed, and Quick-DNA/RNA miniprep plus kit (Zymo) was used to recover both DNA and RNA. DNA was used then to examine the 29c promoter region, and the amplification was presented relative to input. RNA was converted to cDNA with iScript CDNA synthesis kit (Bio-Rad, cat no 1708890). For IPW qPCR, 5 s RNA was used as internal control.

### Western blot

Cell lysate was prepared using RIPA buffer, and debris was removed by centrifuging at 10,000 RPM for 10 min. The lysate was then quantified in NanoDrop 2000, ran on 10% SDS-polyacrylamide gel and then transferred to nitrocellulose membrane (Bio Rad) by the semi-dry method (16 V for 3 h). Membrane was blocked by 5% nonfat skim milk powder dissolved in PBST for 30 min at room temperature. Blots were incubated with ID2 (1:500, Abcam), GAPDH (1:5000, CST), OCT4 (1:1000, CST), SOX2 (1: 1000, CST), NANOG (1:1000, CST), p21 (1:1000, CST) primary and anti-rabbit (1:2000, CST) and mouse (1:2000 Sigma) HRP-conjugated secondary antibodies. Primary antibody was incubated overnight at 4^0^C, while secondary antibody was incubated for 1 h at room temperature. Blots were developed with the use of ECL western blotting detection reagent (GE healthcare, RPN2236) and Amersham Imager.

### Immunohistochemistry

Tumor tissues were isolated from mice at the experimental endpoint and formalin-fixed paraffin-embedded, and 5-mm tissue sections were prepared. Deparaffinization was carried out by heating the slides in oven at 100^0^ C for 30 min followed by rehydration in series on xylene and ethanol. Antigen retrieval was done in sodium citrate (10 mM, pH 6) buffer for 95 °C for 30 min. Endogenous peroxidase was quenched by treating the slides with 3% H_2_O_2_ for 15 min at room temperature followed by PBS washes, 3 × 5 min. Afterward, slides were washed in PBS and blocked with 2% BSA for 1 h followed by incubation with ID2 antibody (1:200, Abcam), OCT4 (1:200,CST), SOX2 (1:200,CST), NANOG (1:200,CST), p21 (1:200,CST), cytokeratin (1:200, abcam) at 4^0^C overnight. This was followed by washing with PBST three times. Then, sections were incubated with secondary antibody and visualized using HRP-based Envision-plus kit (Dako Corp.). The tumor sections were ranked based on staining intensity to calculate the percentage of positive cells.

### Animal experiments

Animal experiments were performed in line with protocol approved by Wake Forest Institutional Animal Care and Use committee. Animals were housed in temperature controlled and pathogen-free environment with 12-h light/12-h dark cycle and free access to food and water. Puromycin-selected DCIS IPW and DCIS pSIN cells were transduced with pSIN luciferase virus. 1 × 10^6^ cells were resuspended in Matrigel and injected in mammary fat pad of nude mice (*n* = 10/group). Animals were then imaged by IVIS every week after injection. For drug treatment, animals (*n* = 6/group) were injected with luciferase-labelled 1 × 10^6^ DCIS.com cells in mammary fat pad. Toyocamycin treatment (2 mg/kg) was started after 2 weeks of cell implantation and administered once a week intraperitoneally. The tumor growth was quantified by IVIS imaging mice twice a week after drug injection. Animals were killed when the tumors in the control group reached 1000mm^3^ or morbid as instructed by the veterinarians. All animals were randomized before the experiment.

## Supplementary Information

**Additional file 1**. Supplementary information.

## Data Availability

Not applicable.
