# Supplementary material for: LncRNA IPW inhibits growth of ductal carcinoma in situ by downregulating ID2 through miR-29c
Source: Breast Cancer Res. 2022 Jan 25;24:6. doi: 10.1186/s13058-022-01504-4 (PMC8787949; doi:10.1186/s13058-022-01504-4)
Supplement: Supplementary file 1 — Additional file 1. Supplementary information. [file 13058_2022_1504_MOESM1_ESM.docx]

**
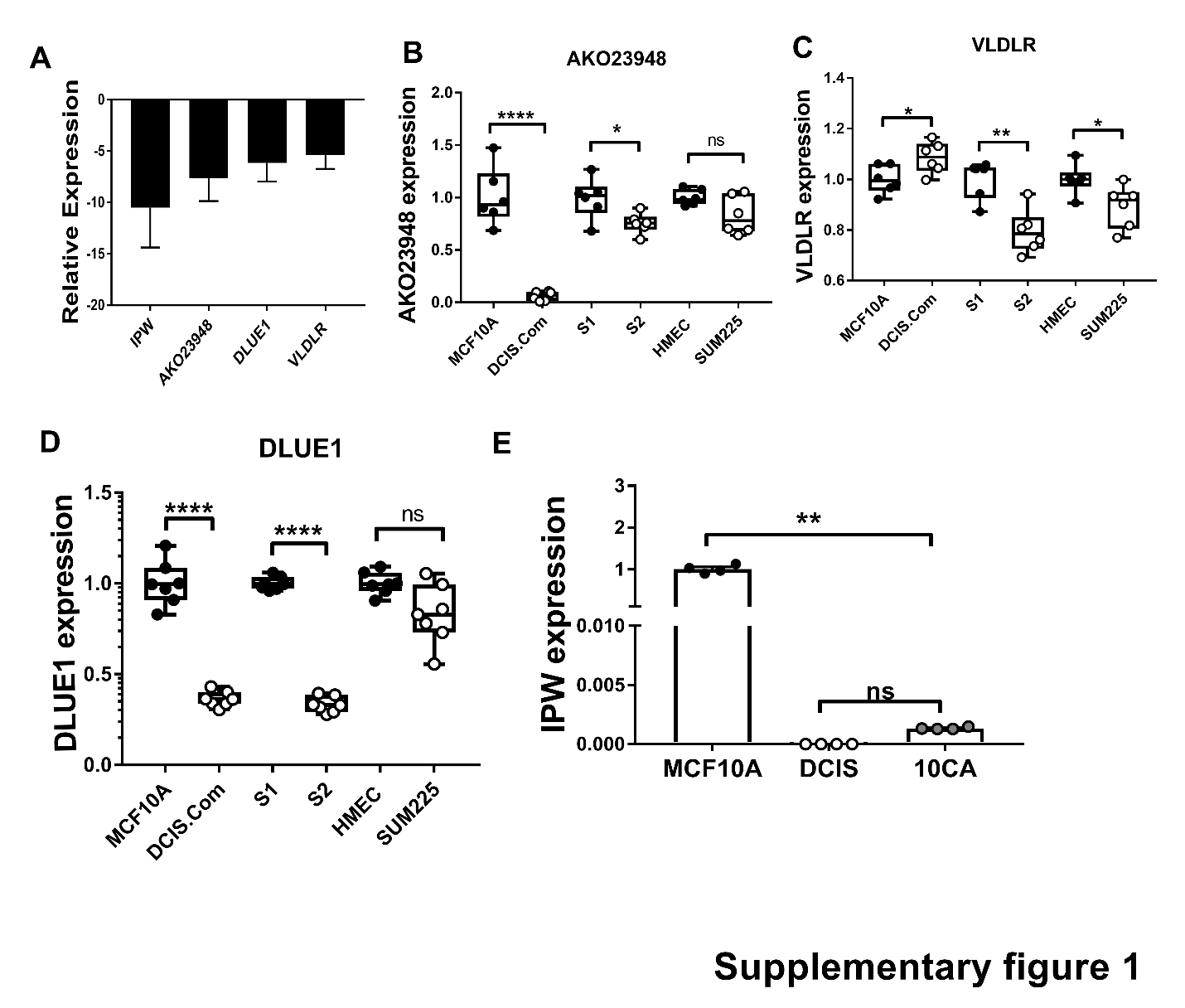
**

**Supplementary figure 1: LncRNA IPW is downregulated in DCIS.**

**A:** Expressions of IPW, AKO23948, DLUE1, VLDLR were examined in paired normal and DCIS samples by qRT-PCR. Relative decrease of lncRNA expression when compared to their expression in DCIS is shown. **B-D:** Expressions of AKO23948 (B), VLDLR (C) and DLUE1 (D) were examined in three pairs of non-tumorigenic and DCIS cell lines - MCF10A and DCIS.com, S1 and S2 and HMEC and SUM225 by qRT-PCR. Unpaired two tailed Student’s t test was performed for statistical comparison. **E:** IPW expression was examined in syngeneic cell lines representing non-tumorigenic (10A), DCIS (DCIS.com) and IDC (10CA) (n=4/group) by qRT PCR. Results were analyzed by one way ANOVA with Tukey post hoc test. All results are represented as mean +S.E.M (*p<0.05, **p<0.01, ****p<0.0001). ns: not significant.

**
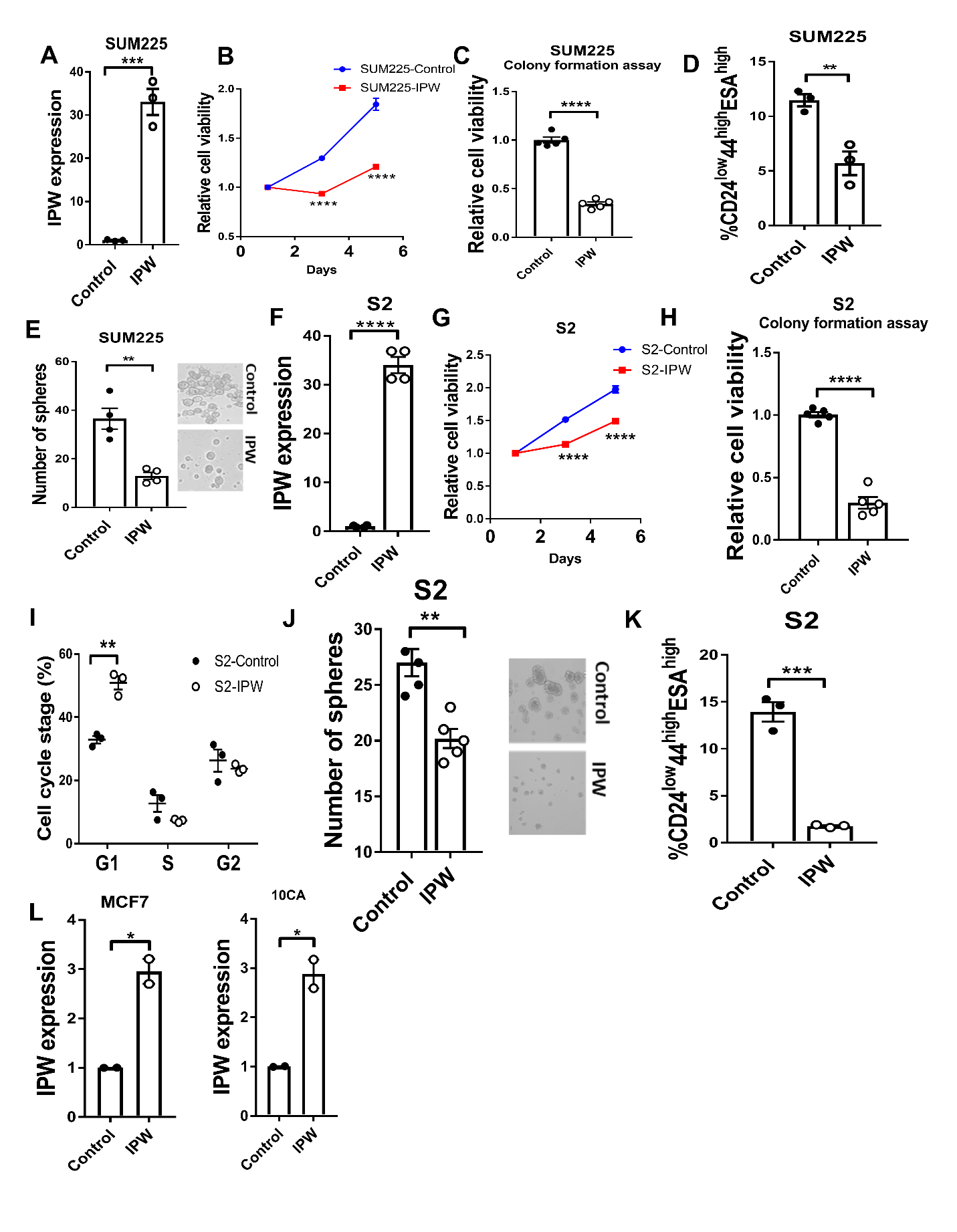
**

**
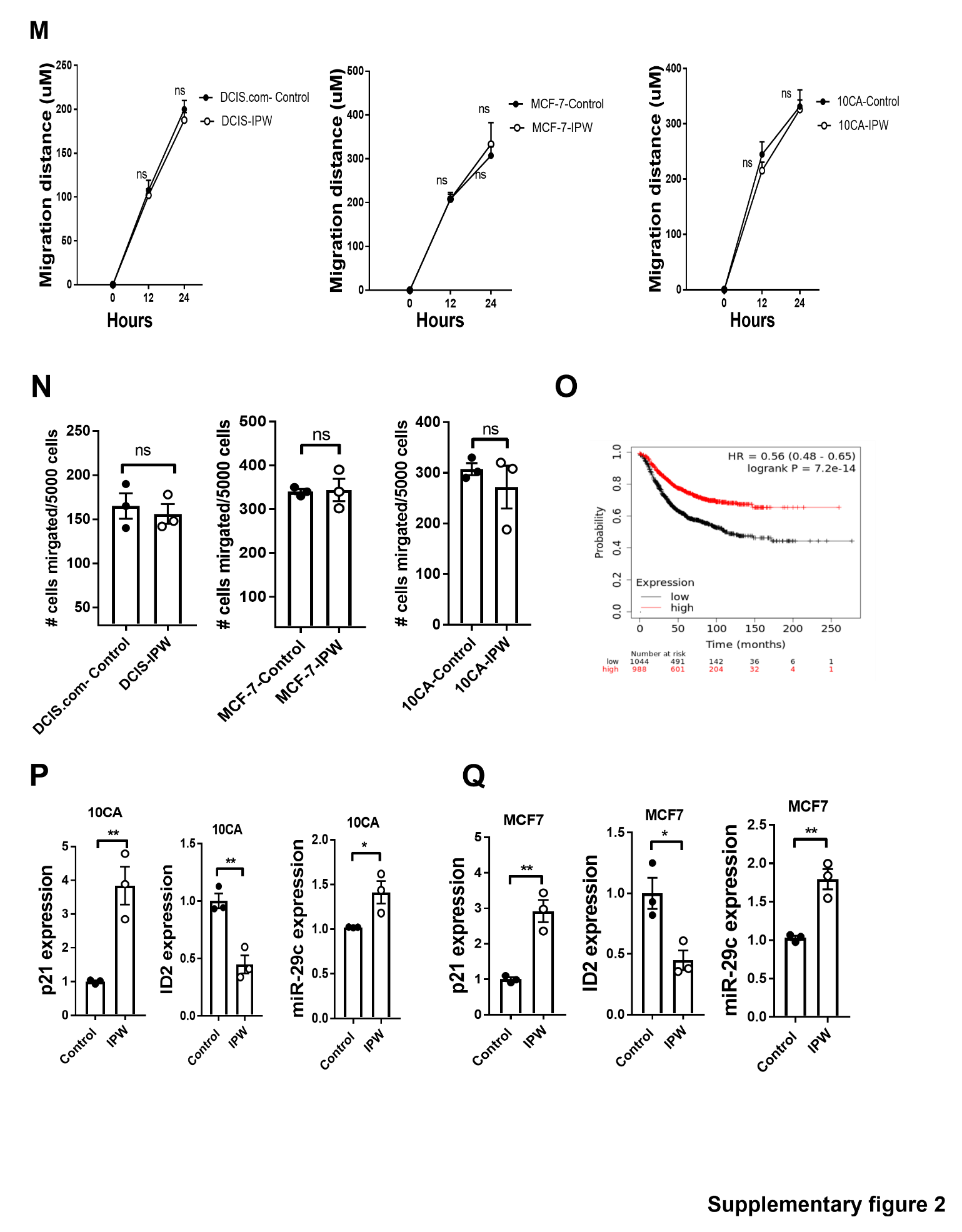
**

**Supplementary figure 2: IPW inhibits DCIS growth in-vitro and in-vivo.**

**A:** LncRNA IPW or empty vector was ectopically expressed in SUM225 cell line using lentivirus, and IPW expression was examined by qRT-PCR (n=4/group). **B:** SUM225 cells transduced with empty vector or IPW. 5000 cells/ well were seeded/well in 96 well plate (n=7/group) and relative cell viability was examined by MTS assay at day 1, 3 and 5. **C:** SUM225-control or SUM225-IPW were seeded in 6 well plates (500 cells/well, n=6/group). The colonies were stained with crystal violet and the dye was dissolved in 10% acetic acid at D10. Subsequently, absorbance at 595 nm was measured by spectrophotometer. **D:** SUM225(control) and SUM225-IPW cells were stained with CD24, CD44 and ESA antibodies subjected to flow cytometry analysis for population of tumor initiating stem cells (CD24low44highESAhigh, n=3/group). **E:** SUM225-pSIN and SUM225-IPW cells were seeded in ultra-low binding plate (1000 cells/well, n=4/group) in mammosphere media. The number of spheres were counted at day 5. The representative image is shown on right panel. **F:** LncRNA IPW or empty vector was ectopically expressed in S2 cell line using lentivirus, and IPW expression was examined by qRT-PCR (n=4/group). **G:** S2 cells were transduced with empty vector or IPW. Cell viability was evaluated by at day 1, 3 and 5 by MTS assay (5000 cells/well, n=7/group). **H:** Colony formation assay was performed by seeding S2-control and S2-IPW cells in 6 well plate (500 cells/well) n=5/group). At D10, the colonies were stained with crystal violet and the dye was dissolved in 10% acetic acid. Absorbance was measured at595nm by spectrophotometer. **I:** S2 (control) and S2-IPW cells were stained with PI and subjected to cell cycle analysis using flow cytometry. % of cells at G1, S and G2 phase are shown (n=3/group). ANOVA and Tukey’s multiple comparison test was done for statistical inference. **J:** S2 (control) and S2-IPW cells were seeded in ultra-low binding plate supplemented with mammosphere media (1000 cells/well, n=4/group). Number of spheres were counted at day 5. Representative images are shown in right panel. **K:** The population of tumor initiating stem cells (CD24low44highESAhigh ) in S2-pSIN and S2-IPW was quantified by flow cytometry (n=3/group). **L:** LncRNA IPW or empty vector was ectopically expressed in MCF-7 (left) and 10ca (right) cell lines using lentivirus, and IPW expression was examined by qRT-PCR (n=2/group) **M:** DCIS.com (left) and MCF-7 (middle) and 10CA (right) cells without and with ectopic IPW expression were grown until confluence in 12 well dish and wound made with blunted tip. Cells were washed with PBS and incubated in culture media without serum for designated time points. The migration distance was calculated by All in one Keyence fluorescence microscope (n=3/group). **N:** DCIS.com (left) and MCF-7 (middle) and 10CA (right) cells with and without ectopic IPW expression were seeded cell culture inserts coated with growth factor reduced matrigel. The migrated cells in lower part were fixed with 4% formalin and stained with crystal violet. Cells were counted with All in one Keyence fluorescence microscope (n=3/group). **O:** Prognostic significance (Relapse free survival) of IPW expression was evaluated through KM plotter in IDC patients. P: Expression of p21 (left), ID2 (middle) and miR-29c (right) was examined in 10CA cells transduced with either IPW expressing or empty vector (control) by qRT PCR (n=3/group). **Q:** Expression of p21 (left), ID2 (middle) and miR-29c (right) was examined in MCF7 cells transduced with either IPW expressing or empty vector (control) by qRT PCR (n=3/group). miR-29c expression was examined by TaqMan probes based qRT PCR, miR-361-5p was used as internal control while for ID2 and p21, beta actin was used as internal control. Statistical inference unless otherwise specified was determined by unpaired two tailed Students t test. Data is represented as mean + S.E.M. (*p<0.05, **p<0.01, ***p<0.001, ****p<0.0001). ns: not significant.

**
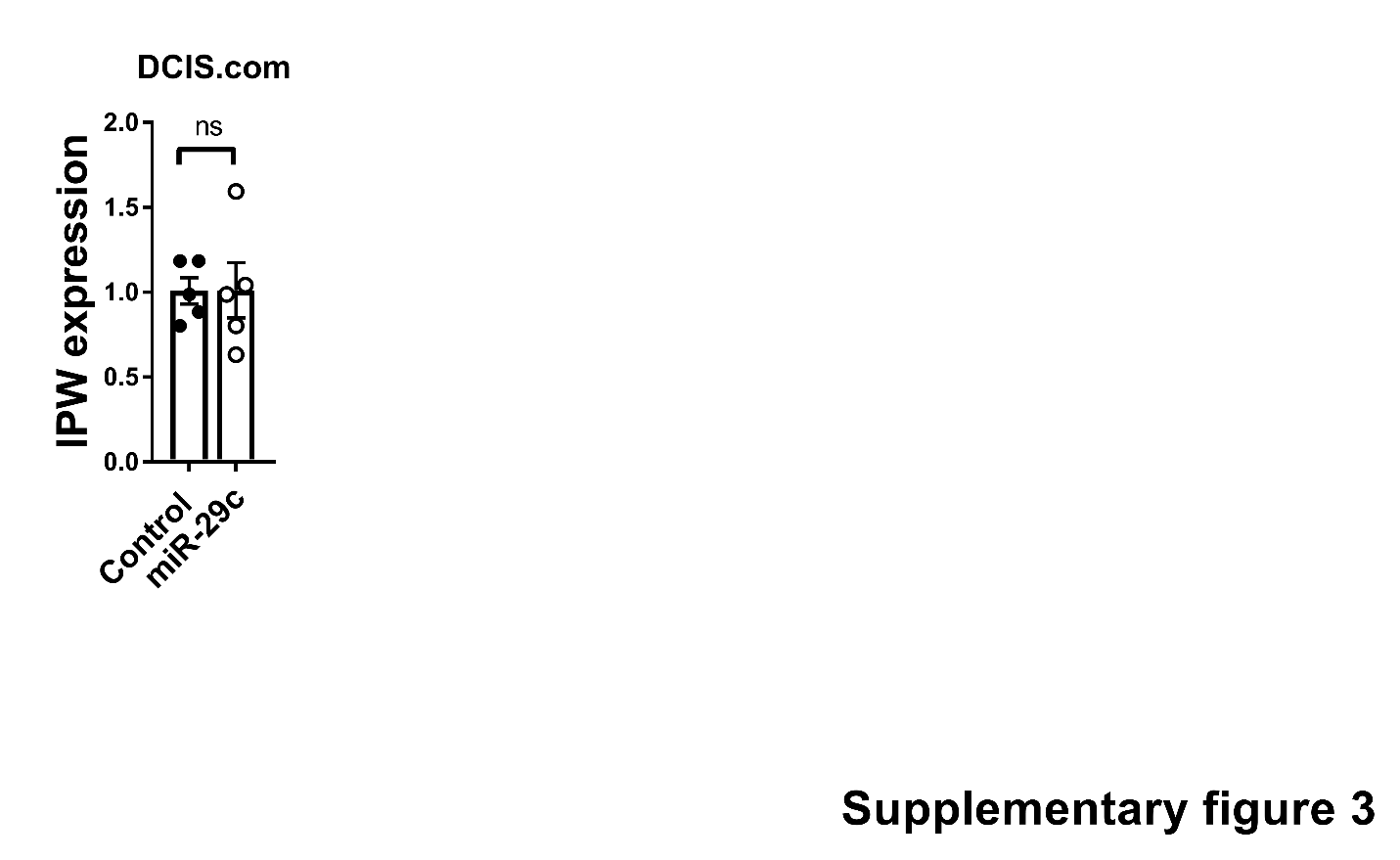
**

**Supplementary figure 3. miR-29c is downregulated in DCIS and is controlled by IPW.**

DCIS.com cells were transduced with either empty or miR-29c expression vector. The expression of IPW was examined by qRT-PCR (n=5/group). Statistical inference was determined by unpaired two-tailed Student’s t test. Data is represented as mean + S.E.M. ns: not significant.

**
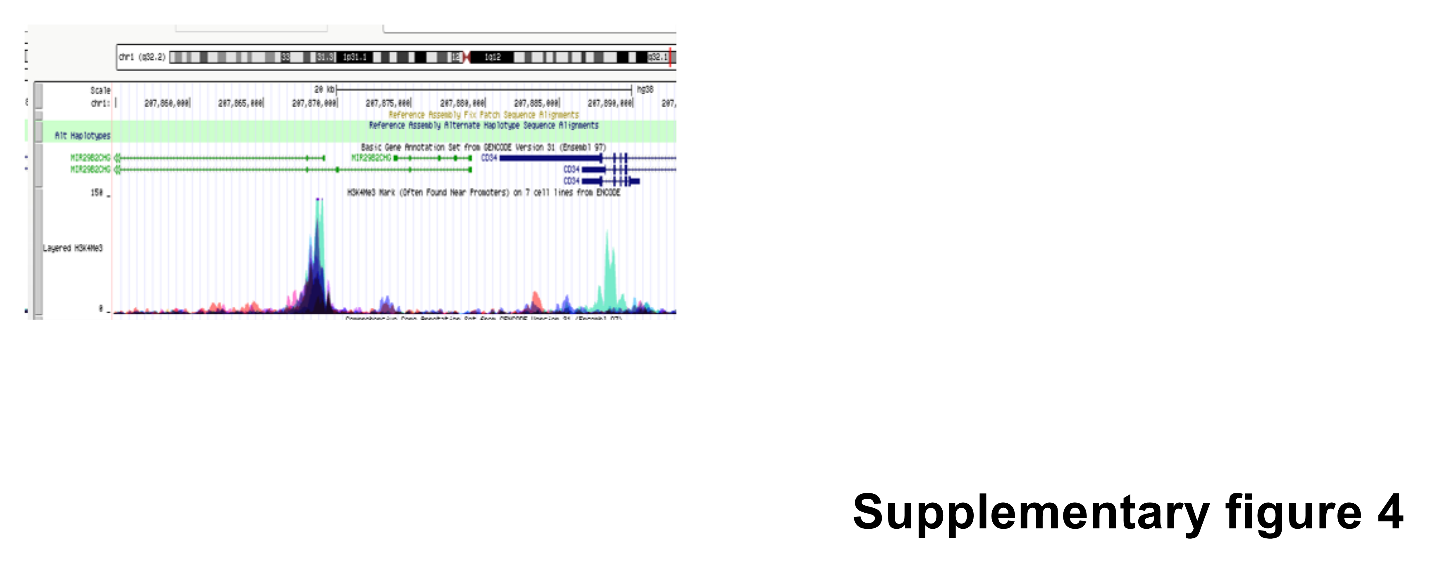
**

**Supplementary figure 4. IPW upregulates miR-29c expression by enhancing H3K4 trimethylation.**

UCSC genome browser map of miR-29c promoter region with H3K4me3 enrichment near to its promoter.

**
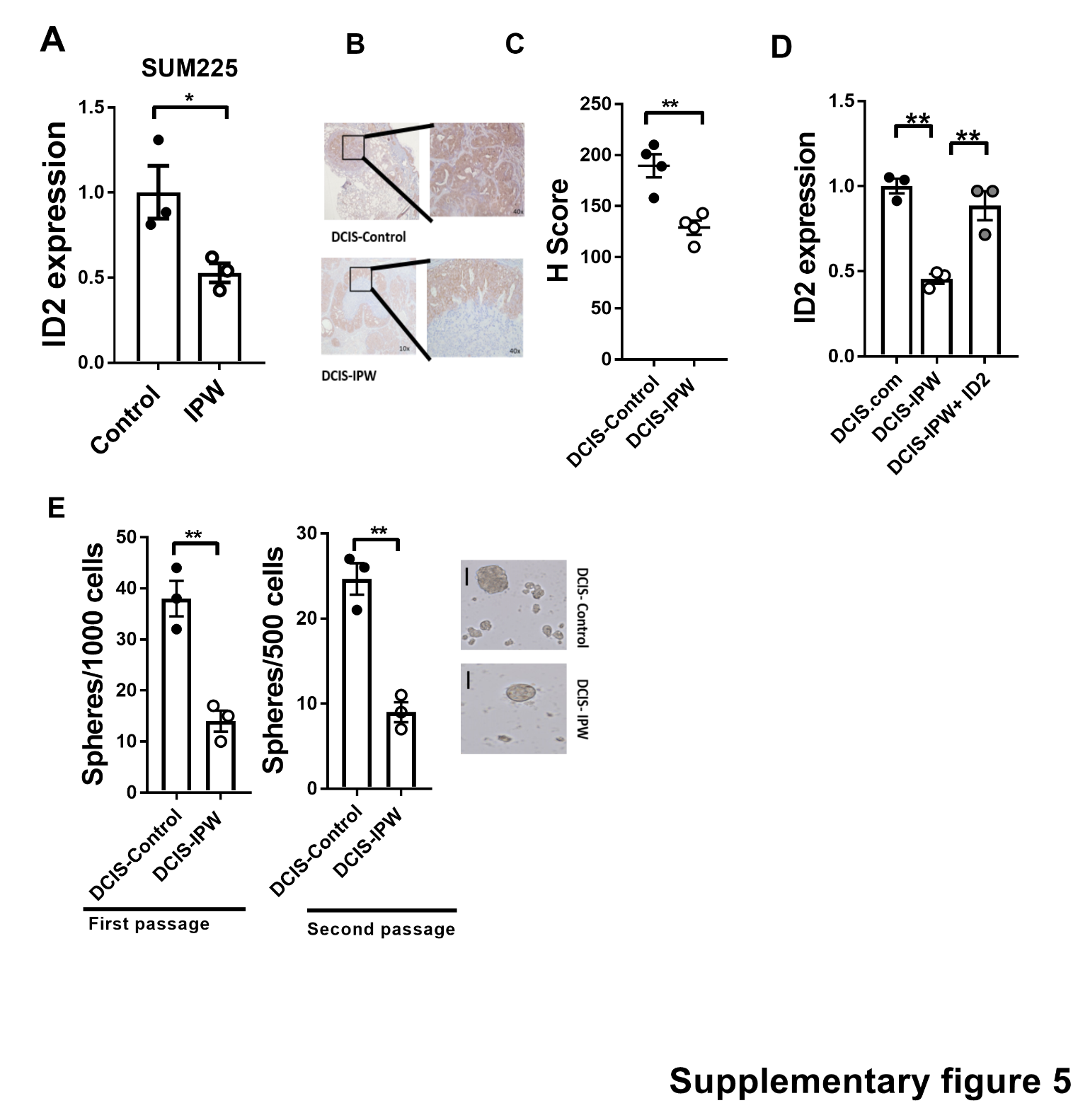
**

**Supplementary figure 5: miR-29c suppresses self-renewal ability of DCIS by targeting ID2.**

**A:** ID2 expression was examined in SUM225 cells transduced with IPW (n=3) by qRT-PCR. Beta actin was used as internal control. Statistical inference was made using unpaired two tailed Student’s t-test and represented as mean + SEM. **B:** Tumor tissues from mouse implanted with DCIS (control) and DCIS-IPW were examined for ID2 expression by immunohistochemistry (IHC). Representative IHC images are shown. **C:** Quantification of ID2 staining in Figure B. **D:** DCIS.com cells were transduced with IPW and ID2 expressing vector. Total RNA was isolated and ID2 mRNA expression was examined by qRT-PCR (n=3/group). Beta actin was used as internal control. **E:** CD24l^ow^CD44^high^ESA^high^-DCIS.com and DCIS-IPW cells were isolated my MACS and seeded in ultra-low attachment plate (1000 cells/well, n=5/group) (First generation, Left). The first generation spheres dissociated and 500 cells were reseeded in ultra-low attachment plate representing the second generation (Right). Number of spheres were counted at Day 5. Scale bar 50 µm. One-way ANOVA with Tukey multiple comparison post hoc test was performed for statistical comparison data is represented as mean + S.E.M. (*p<0.05, **p<0.01).

**
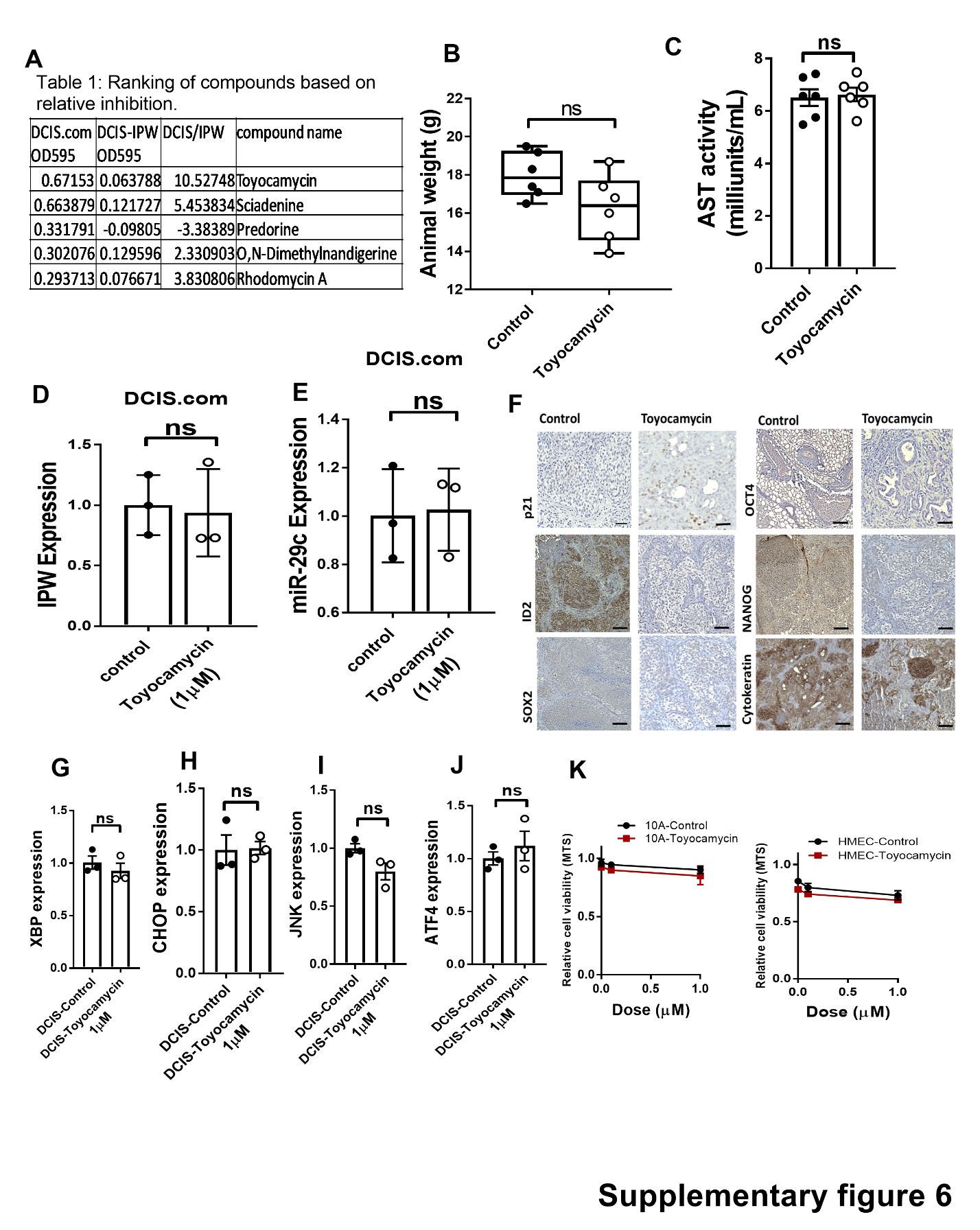
**

**Supplemental figure 6: Toyocamycin selectively inhibits low IPW expressing DCIS.**

**A**: Compounds indexed on the basis of relative inhibition in DCIS.com cells treated with toyocamycin from Figure 6A. Relative inhibition was calculated by formula Control-test/control x100. **B:** Toyocamycin or control (DMSO) treated animals from Figure 6D were measured for weight at end the point. **C:** Serum AST levels were measured in the serum from control and toyocamycin treated animals from Figure 6D at the end point to examine liver toxicity (n=6/group). Statistical inference between groups unless otherwise specified was determined by unpaired two tailed Student’s t test and data is represented as mean + S.E.M. **D:** DCIS.com cells treated with either control (DMSO) or toyocamycin (1µM) for 24h followed by RNA isolation. IPW expression examined by qRT-PCR (n=3/group). 18s rRNA was used as internal control and miR-29c **(E)** expression was examined by TaqMan-based qRT-PCR (n=3/group). miR-361-5p was used as internal control. **F:** p21, ID2, OCT4, SOX2 and NANOG protein expression was examined from the FFPE sections prepared after animals were sacrificed at end point from Figure 6D. Sections also stained with human cytokeratin to confirm that the tumor tissues derived from implanted cells. Scale bar 100 µm. **G:** DCIS.com cells were treated with either control (DMSO) or toyocamycin (1µM). Total RNA was isolated and expression of selected genes as XBP, **H:** CHOP, **I:** JNK, **J:** ATF4 having role in ER stress response were examined by qRT PCR. Beta actin was used as internal control (n=3/group). **K:** MCF-10A (left) and HMEC (right) cells were treated with toyocamycin or control (DMSO) at indicated concentrations for 48h and relative cell toxicity was evaluated by MTS assay (5000 cells, n=3/group). Statistical inference between two groups was determined by unpaired two tailed Student’s t test and data is represented as mean + S.E.M. (*p<0.05, **p<0.01, ***p<0.001, ****p<0.0001).ns: not significant.
